# Phylogenetic signals in pest abundance and distribution range of spider mites

**DOI:** 10.1186/s12862-019-1548-3

**Published:** 2019-12-05

**Authors:** Peng-Yu Jin, Jing-Tao Sun, Ary Hoffmann, Yan-Fei Guo, Jin-Cheng Zhou, Yu-Xi Zhu, Lei Chen, Xiao-Yue Hong

**Affiliations:** 1 Department of Entomology, Nanjing Agricultural University, Nanjing, 210095 Jiangsu China; 2 School of BioSciences, Bio21 Institute, The University of Melbourne, Melbourne, Victoria Australia; 3 School of Plant Protection, Shenyang Agricultural University, Shenyang, 110866 Liaoning China

**Keywords:** Pest occurrence, Phylogenetic signal, Host range, Spider mite

## Abstract

**Background:**

Attributes of pest species like host range are frequently reported as being evolutionarily constrained and showing phylogenetic signal. Because these attributes in turn could influence the abundance and impact of species, phylogenetic information could be useful in predicting the likely status of pests. In this study, we used regional (China) and global datasets to investigate phylogenetic patterns in occurrence patterns and host ranges of spider mites, which constitute a pest group of many cropping systems worldwide.

**Results:**

We found significant phylogenetic signal in relative abundance and distribution range both at the regional and global scales. Relative abundance and range size of spider mites were positively correlated with host range, although these correlations became weaker after controlling for phylogeny.

**Conclusions:**

The results suggest that pest impacts are evolutionarily constrained. Information that is easily obtainable – including the number of known hosts and phylogenetic position of the mites – could therefore be useful in predicting future pest risk of species.

## Background

The human modification of natural environments including expansion of agricultural production areas has been a primary driver of terrestrial biodiversity loss [10]. Although hundreds of species have been documented as dramatically declining under habitat modification, some species are thriving [44], including agricultural pests and pathogens, which in turn have led to additional stresses on non-pest species [28]. Understanding why some species fare poorly whereas others do well has been a key issue of concern to biologists, ecologists, agriculturists and policymakers [46, 61], and is an important consideration when assessing future risks of species extinctions as well as pest outbreaks [58].

Species extinction risk is often not randomly spread across phylogeny [3], indicating that phylogeny could be useful in predicting the fate of species [19, 52]. In risk assessments, phylogenetic information has also been used to predict which plant species are likely to be susceptible to a particular pest [23, 55], because closely-related plants tend to have similar traits (e.g. plant defensive chemicals) and host similar pests when compared to evolutionarily distant plant species [24]. However, the host plant records for many novel pests are incomplete, and the severity of pest outbreaks may not be closely linked to this factor; for instance, information of pest host range and host phylogeny was insufficient to determine whether pests on a given host or novel region were severe or benign [23].

Predicting potential risks posed by a pest or pathogen requires an understanding of a range of biological and ecological characteristics for adapting to particular hosts and agricultural contexts [11, 16], as well as an assessment of the degree to which these are constrained within phylogeny [35, 66]. Species’ performance is often determined by traits that show a strong tendency to take similar values among closely related species [18], including host plant range [24] and thermal resistance [35]. These traits in turn are likely to alter the demographic characteristics of species and link to species distribution and abundance ([25, 32]) which in turn are expected to be phylogenetically structured. Phylogenetic distance between poorly-known novel pests and well-studied pest species with known occurrence pattern may therefore help predict whether a novel pest is likely to have severe effects.

To test these ideas, we examined phylogenetic patterns of pest occurrence in a phytophagous species group, the spider mite family (Acari: Tetranychidae). This family includes more than 1300 species (around 100 of which are considered pests) that share similar morphological characters but vary in host breadth (extremely polyphagous vs. highly host-specific) and distribution range (widespread vs. narrow distributions) [45]. These features make spider mites a useful group to investigate host- and distribution-related ecological and evolutionary questions [63, 64], although most previous work has focused on the intra-specific level rather than the comparative level. We therefore aim to test for phylogenetic signal in relative abundance and host range and link findings to pest outbreak patterns. This work can guide phytosanitary risk analysis of pests and their potential impact before pests arise in a region [25, 54].

Here we used long-term survey data from 2008 to 2017 in China and species information from a global dataset to test for phylogenetic signatures in spider mites. Our analysis addresses three main questions. First, are the host range and relative abundance of spider mites non-random within phylogeny? Second, does host range correlate with species occurrence patterns? And third, how does phylogeny influence the host range - abundance relationship?

## Results

### Phylogenetic signal of pest occurrence

**Fig. 1:**
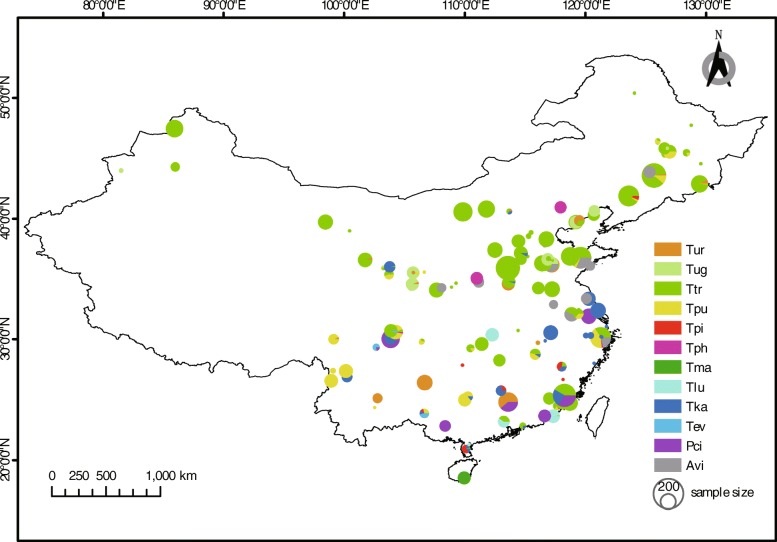
Sampling information in China. All spider mite sample derive a long filed survey from our lab. Sample sites with different population size and species composition structure were plotted on a base map using ERSI ArcGIS (ArcMap 10.2.2, Redlands, CA, USA). Circles with different colors represent the species composition at each site. Circle size represents the sample numbers at each site., Abbreviations: Tur, *Tetranychus urticae* (red form); Tug, *T. urticae* (green form); Ttr, *T. truncatus*; Tpu, *T. pueraricola*; Tpi, *T. piercei*; Tph, *T. phaselus*; Tma, *T. macfarlanei*; Tlu, *T. luden*i; Tka, *T. kanzawai*; Tev, *T. evansi*; Pci, *Panonychus citri*; Avi, *Amphitetranychus viennensis*

In our field survey, twelve spider mite taxa were found from 318 populations at 180 sites (Fig. 1, also see Additional file 1: Table S1), which included 7596 samples. Ten species of spider mites belonged to *Tetranychus*, one species belonged to *Panonychus* and one species belonged to *Amphitetranychus* (Fig. 1, also see Additional file 1: Table S3. The two measures of mite abundance (NOC and AF) were strongly and positively correlated (Pearson *r* = 0.996, *p* <  0.001). High correlation coefficient (Pearson *r* = 0.959, *p* <  0.001) also was found between two measures of host range (host species and host family). We therefore only considered NOC and host family in the analyses.

Ten of 12 species occurring in this survey belonged to the genus *Tetranychus*, and the molecular distance between these species was compared to the pattern of occurrence and host range in China. Phylogenetic trees used for phylogenetic signal tests were reconstructed with the BI and ML methods (Additional file 1: Figure S2 in ESM). The BI and ML trees of the combined three DNA fragments (COI, 18S and 28S) resulted in identical topologies for both China dataset (Additional file 1: Figure S1) and global dataset (Additional file 1**:** Figure S2, S3). In both cases, the topology is mostly well supported, with a bootstrap support value and posterior probability value in major nodes. The phylogenies were corresponding with prior phylogenies [43]. No significant correlation between relative abundance and genetic distance was detected using a Mantel test (Additional file 1: Table S4, *p* = 0.119). However, the phylogenetic signal for species abundance (across all 10 species) [log_10_ (NOC)] was higher than random expectations and larger than expected under a Brownian motion model of character evolution (K = 1.032, *p* = 0.033) and a significant phylogenetic signal was also detected using Abouheif’s test (*p* = 0.013). The species distribution range size (latitudinal span) showed significant phylogenetic signal on all three measures (Table 1). For the global dataset, weak phylogenetic signal was detected in occurrence patterns (historical records number and distributed country number) with all three measures (Table 1).

**Table 1:** Phylogenetic signal analysis for species abundance, distribution range and host range of spider mite

		Blomberg’s K		Abouheif’s C	
		K	*p* value	C	*p* value
China	Relative abundance	1.032	0.033	0.405	0.013
	Host range	1.263	0.009	0.395	0.025
	Latitudinal span	1.708	0.001	0.500	0.005
Global	Records number	0.071	0.001	0.224	0.001
	Host range	0.075	0.001	0.207	0.001
	Number of distributed country	0.050	0.014	0.118	0.001

Blomberg’s K [6] and Abouheif’s C test [1] are two measures of phylogenetic signal. Traits with probabilities < 0.05 were considered to have significant phylogenetic signal. Higher C/K value indicate stronger phylogenetic signal

**Fig. 2:**
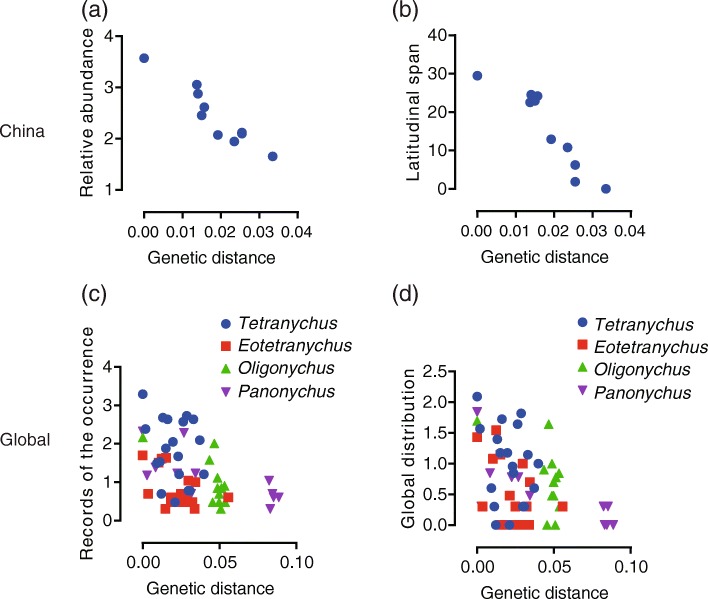
Relationships between the species occurrence patterns and genetic distance to the focal species. **a** Genetic distance vs. relative abundance (total number of occurrence) for China dataset; **b** Genetic distance vs. latitudinal span for China dataset; **c** Genetic distance vs. records number of occurrence for global dataset; **d** Genetic distance vs. distribution for global dataset. Values of relative abundance, global records number of occurrence and global distribution (number of countries) were log transformed. Different genara are indicated by different colours and symbols

For *Tetranychus* species in China, the relative abundance (Fig. 2a, *r* = 0.943, *p* <  0.001) and distribution range (Fig. 2b, r = 0.924, *p* <  0.001) of species declined significantly with increasing phylogenetic distance to focal species based on a correlation analysis. Such a pattern also existed in the global dataset (Fig. 2c, d). For the global dataset, the correlation coefficients and their significance were different among the genera (Additional file 1: Table S5). Three of four tested genera (*Eotetranychus*, *Oligonychus* and *Panonychus*) showed significant correlations between occurrence patterns (historical records number and distributed country number) and genetic distance to the focal species for the global dataset.

### Relationships between host range and species occurrence

**Fig. 3:**
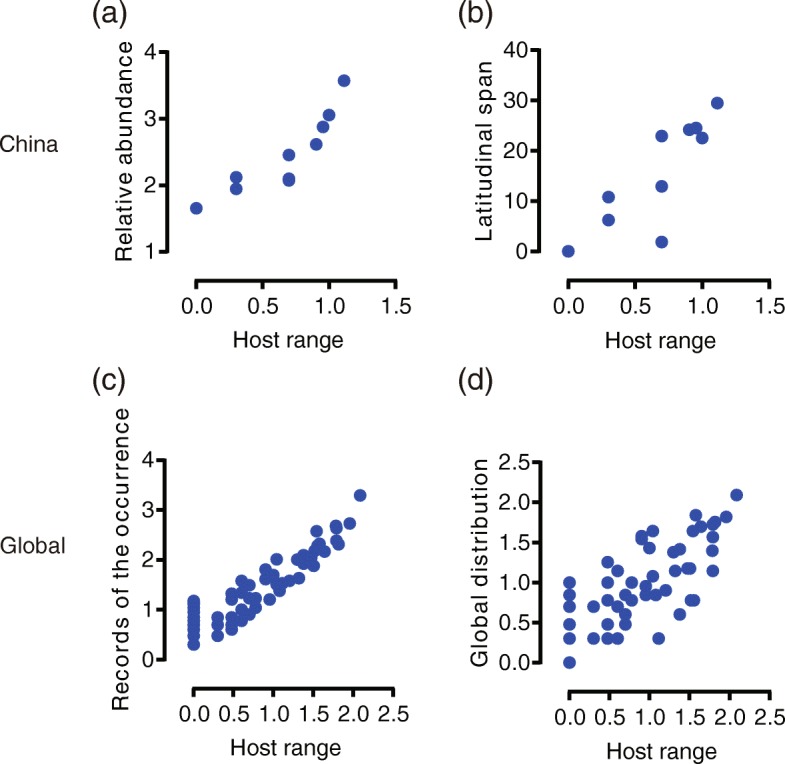
Relationships between the host range (host family number) and species occurrence patterns. **a** Host range vs. relative abundance (total number of occurrence) for China dataset; **b** Host range vs. latitudinal span for China dataset; **c** Host range vs. records number of occurrence for global dataset; **d** Host range vs. global distribution (number of countries). Values of relative abundance, host range, global records number of occurrence and global distribution were log transformed

For species in China, the relative abundance and latitudinal range of spider mites were significantly associated with host range (Fig. 3a, b). To investigate whether phylogeny influences the host range – species occurrence relationship, we performed phylogenetically corrected correlations between host range and pest occurrence. The relationships between host range and pest occurrence tended to become weaker with lower coefficients after PIC and PGLS correction for phylogeny (Table 2). Similar patterns were also found in the global dataset (Fig. 3c, d). Although significant correlations between host range and pest occurrence remained, the strength of all relationships was reduced by phylogenic correction (Table 2).

**Table 2:** Correlations between host range and pest occurrence using Pearson’s correlations, phylogenetically independent contrasts method (PIC) and phylogenetic generalized linear model (PGLS)

		Pearson		PIC		PGLS	
		r	*p* value	r	*p* value	r	*p* value
China	Host - abundance	0.883	0.001	0.849	0.004	0.808	0.003
	Host - distribution	0.833	0.003	0.710	0.031	0.754	0.007
global	Host - abundance	0.939	< 0.001	0.885	< 0.001	0.894	< 0.001
	Host - distribution	0.855	< 0.001	0.831	< 0.001	0.839	< 0.001

**Fig. 4:**
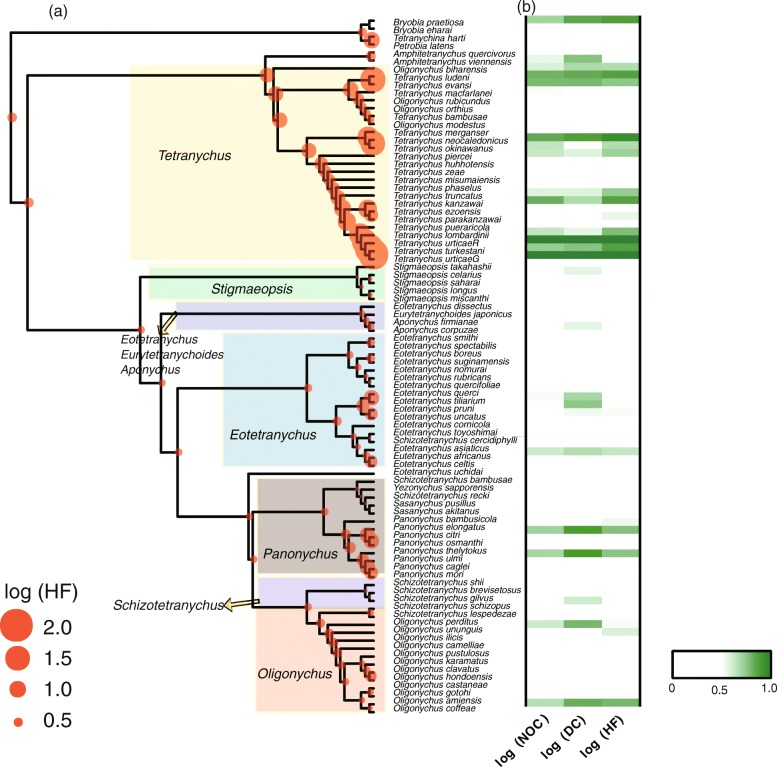
Phylogeny of Tetranychidae, and species occurrence and host range a global scale. **a** Phylogenetic tree inferred from three combined DNA fragments (COI, 18S and 28S) in RaxmlGUI1.3. The circles shown next to the branches are from the ancestral trait reconstruction calculated using maximum likelihood methods for host famliy number (HF). Values (log transformed) were represeented by the circle size; **b** Heatmap of occurrence of records number (NOC), number of distributed countries (DC) and number of host families (HF). Data were log transformed and scaled to the 0–1 range for organizing the heat map

The ancestral trait reconstruction showed different patterns of host range evolution among clades at the genus and subgenus levels (Fig. 4). This analysis suggested a monophagous origin of spider mites. The ancestral state of narrow host range seems persist in other clades within the evolutionary history of spider mites. But host range expanded rapidly in the clade *Tetranychus,* and the evolution of host range expansion was mostly restricted to this group. Several species in other groups also had a wide host range (e.g. *Oligonychus coffeae*), yet most maintained a narrow host range as for the ancestral form.

## Discussion

We found strong phylogenetic signal in pest abundance and distribution of *Tetranychus* mites at a regional scale (China), and also detected a phylogenetic signature for species occurrence when analyzing 88 spider mite species using a global dataset. Pest occurrence (relative abundance and distribution range size) declined predictably with increasing genetic distance from the most abundant pest species. These results suggest that species occurrence can be partly predicted by evolutionary relationships in the spider mite group.

Several studies have introduced phylogenetic information into pest risk assessments [22, 23]. However, such information was not sufficient to evaluate whether pest damage on a given host or in a novel region is severe or benign [23]. Here we tested another hypothesis, namely that the phylogeny of pests themselves can be used to predict which pest species are likely to be abundant.

The evolutionary history of pests can significantly affect their capacity to adapt to new host plants or novel environments [13, 50], leading to a potential relationship between pest severity and phylogeny. This was confirmed in spider mites; there were significant phylogenetic signal in both relative abundance and distribution range size. Many species belonging to the genus *Tetranychus* were relatively common and also were serious pests locally (e.g. *T. truncatus* in China) [33] and with the potential to become global pests. For example, *T. urticae* and *T. evansi* have expanded their distribution and become serious pests in many regions [7, 26, 62]. However a categorical metric of pest risk decision process, (e.g., all pest within a genus are risky, and others are not) is not ideal for risk analysis of novel pests and pathogens [23]. Because we found that the relative abundance and distribution range size declined as a function of phylogenetic distance between congeneric spider mites, we suspect that species relatedness data within genera may be useful in pest risk assessments in the absence of other empirical information.

In this study, we detected strong and positive correlations between host range and relative abundance at both the country and global scales. Species niche breadth is often considered to reflect an evolutionary trade-off between a species’ ability to exploit a wide range of resources and the effectiveness of exploitation [9, 27], resulting in a lower abundance of species exploting a broader host range [8, 65]. However, there are other theories [e.g. the hierarchical theory posed by Passy [49]] arguing that species with the highest maximum abundance and regional prevalence possess the broadest niches, especially under a stressful environment (e.g. short resource supply, human impact). The applicability of these hypotheses may reflect the degree of disturbance in the environment, perhaps caused by human-associated changes (e.g. agriculture and urban expanding, polluting or global warming) [4, 5]. This may help explain why generalists seem to benefit from global change more than specialists [41].

In this study, there was some evidence for a lower abundance of specialists. When we analyzed each host individually, there were diverse relationships (positive, negative or none) between abundance and specialization. When we considered all paired values for the two datasets (12 species in China and the global dataset), there was no significant association for the China dataset (see Additional file 1: Figure S4a). However, for the global dataset, we found negative correlations between host specialization and species occurrence on specific hosts (see Additional file 1: Figure S4b). This runs contrary to the expectation of a trade-off between species niche breadth and performance on a particular host. In insect herbivores, a global scale study showed that more diverse lineages of plants support assemblages of relatively more specialized herbivores [15]. This suggests a lower abundance of specialized herbivores could relate to reduced plant diversity. However, positive host range –abundance relationships in spider mites may reflect the fact mites with a wider niche breadth can reproduce and persist in agricultural ecosystems on a greater range of crops and therefore build up across time.

The moderate to high phylogenetic signal in host range at both regional and global scales suggests non-random evolution of host range in spider mites. Two phylogenetically corrected correlation analyses (PIC and PGLS) showed that relationships tended to become weaker with lower coefficients after correction for phylogeny. This finding was supported by the ancestral trait reconstruction analysis (Fig. 4) showing that the evolutionary pattern of host range was different among clades. The majority of clades showed relative conservative patterns in host range, whereas host range in some clades have rapidly expanded after an early split with others. In particular, the frequency of evolutionary expansion in host range appears to increase dramatically at the *Tetranychus* group. Such patterns indicate evolutionary history is important to understanding species’ status in community [30, 67].

Compared to local scale, we found host range and occurrence showed lower phylogenetic signals (Table 1). The association between species occurrence and genetic distance also tend to be weaker at global scale (Fig. 2). However, the association between species occurrence and host range tend to be stronger at global scale than at local scale (Table 2). These results suggested host range maybe more relevant than phylogenetic signals on predicting pest risk at global scale.

False positive correlations can be produced across species comparisons, including scale selection [69], sampling effects [38] and statistics [68]. The different strength and significance level of phylogenetic signal between regional and global scale suggests phylogenetic patterns could be influenced by sampling issues [39]. In this study, only 88 of 1300 species were used for phylogenetic analysis, although the most comprehensive tree was developed based on available data [43]. Further analysis of additional species (and particularly rare species) may provide insights into how species abundance and niches are distributed across subgeneric-level phylogenies. For tests of the host range – pest occurrence relationship, sampling may generate a positive relationship [21]. For our data from China, we suspect that these issues are not likely to obscure patterns. The occurrence data were derived from a long-term survey, and the survey locations covered all major regions of China.

## Conclusions

In summary, we found pest abundance/distribution and host range showed significant phylogenetic signal. Relative abundance and geographic range size of spider mites were positively correlated with host range. These results suggest that phylogenetic information could help to understand the community assembly of this pest group from an evolutionary perspective. Information that is easily obtainable – including the number of known hosts and phylogenetic position of the mites –may contribute to risk analyses of pest outbreaks.

## Methods

### Distribution, relative abundance and host range for species from China

We collected spider mites during the summers of 2008 to 2017 across major regions of China (Fig. 1, also see Additional file 1: Table S1 in ESM). Since the number of spider mites at a collection site is affected by many local factors including pesticide application, host type and sample period [20], the total abundance of mites is expected to differ even among nearby sites. In contrast, the species composition of spider mites at a larger scale is relatively stable [31]. We therefore focused on surveys of multiple sites and estimated relative abundance based on occurrences across sites rather than resampling each site multiple times [33]. At each site (around 3000 m^2^), our strategy was to collect a maximum of three mites per plant, with plants separated by a minimum of 1 m. Overall, 318 geographic or host-associated populations were collected from 180 sites that spanned the native range of spider mites, from Northeastern China to Southwestern China (Fig. 1). As a metric of range size, we calculated the latitudinal span covered by each species [2]. Relative abundance for each species was represented by the total number of occurrences (NOC) in our survey of 318 populations. To minimize any bias associated with intensive sampling in one site, average frequency of occurrence (AF) across different sites was calculated as a second index of mites abundance.

In general, monophagous species tend to feed on a single plant species, oligophagous species tend to feed on one genus and polyphagous species feed on at least one family of plants [57]. Some spider mite species (e.g., *Tetranychus evansi)* can feed on more than 300 host species, but most hosts belong to one family [47]. Other mites may have very few host species, but the hosts belong to more than one host family [45]. We therefore used both host plant species records and host plant family records to represent host range. The host records for each species were obtained from the survey.

### Distribution, occurrence and host range for species from a global dataset

Spider mites web (http://www1.montpellier.inra.fr/CBGP/spmweb/) is a global database which includes host records, distribution countries and historical records for more than 1300 spider mites species [45]. The frequency of occurrence for each species at a global scale was indirectly counted as the number of historical records that had clear host information on Spider mites web [45]. This method may overestimate or underestimate the abundance of a mite species in the wild because of a likely focus on economically important species, but it is still likely to reflect the relative abundance of species within agricultural settings. Host range at a global scale was represented by host family number, which was derived from Spider mites web [45]. Most of the species lacked detailed location information, and the distribution range for each species was estimated from the number of countries where each species was found.

### Phylogeny and phylogenetic signal analyses

The 18S gene, 5′ end of the 28S rRNA gene and mitochondrial COI gene were used for phylogenetic analyses [43] (for GenBank accession ID, see Additional file 1: Table S2 in ESM). Phylogenetic trees were constructed using maximum likelihood (ML) and Bayesian inference (BI) methods following the protocols described by Xue et al. [70]. BI analyses were performed with MrBayes 3.2.2 [56], and two independent runs were conducted, each with four Markov Chains (one cold chain and three heated chains). GTR + I + G was the model chosen by jModelTest 2.1.1 [12]. ML analyses were performed using the GTRGAMMAI model in raxmlHPC-PTHREADS [60] implemented in raxmlGUI1.3 [59]. Genetic distances between species were calculated in MEGA7 [40] applying the Kimura 2-parameter model [37], with 1000 bootstrap replicates.

To test whether species occurrence (relative abundance and distribution) and host range were non-randomly associated with genetic similarity between the species, we used Mantel tests to compare these characteristics with a genetic distance matrix [42] in R-3.4.4 for Windows (R [53]). To quantify the phylogenetic signal of species characters, we computed Blomberg’s K [6] in the package ‘picante’ [36] and Abouheif’s test [1] in package ‘adephylo’ [34]. Both tests were performed in R-3.4.4 for Windows [53]. Blomberg’s K quantifies the amount of phylogenetic signal in the data relative to a Brownian motion model of trait evolution. K = 1 corresponds to a Brownian motion pattern and K = 0 corresponds to a random distribution of the trait across the phylogeny. The higher the K statistic, the more phylogenetic signal in a trait. Traits with PIC.variance probabilities < 0.05 have significant phylogenetic signal. PIC.variance probabilities is the quantile of the observed phylogenetically independent contrast variance versus the null distribution, which can be used as a one-tailed *p*-value to test for greater phylogenetic signal than expected [36]. Abouheif’s test for serial independence is based on the sum of the successive squared differences between trait values of neighboring species [1]. Traits with probabilities < 0.05 were considered phylogenetically structured.

To test whether we could use phylogenic distance to predict pest occurrence for each genus, we first identified the most abundant species as focal species – the species for which the measured response was strongest [22]. Then, we calculated the genetic distance between the focal species and other spider mites, respectively. The relationships between species occurrence (abundance and distribution) and phylogenetic distance were investigated using Pearson correlation analyses.

### Host range and pest occurrence relationships

To test for relationships between host range and species occurrence, we computed correlations between these variables (Pearson’s r). The PGLS (Phylogenetic generalized linear models) [17] function in the ‘caper’ package [48] and PIC (phylogenetically independent contrasts function) [14] in the ‘ape’ package [51] were then used to calculate phylogenetically-corrected correlation coefficients for host range and abundance accounting for variable levels of phylogenetic signal. Both programs provide a phylogenetically corrected r value giving an estimation of the association between the host range and abundance variables following correction for phylogeny. To illustrate how host range evolved within the evolutionary history of spider mites, we reconstruct ancestral states for host range using a maximum likelihood approach based on a BM model in the ‘geiger’ package [29]. Data (Relative abundance, host range and global distribution countries number) were log-transformed to meet requirements for normality in all analyses.

## Supplementary information

**Additional file 1:** **Table S1.** Sample information for samples from China used in this study. **Table S2.** GenBank accession of sequences used in phylogenetic reconstruction at global scale. **Table S3.** Relative abundance, host range and distribution of each spider mite species in China. **Table S4.** Mantel tests of association between genetic distance and species abundance, distribution range and host range. **Table S5.** Pearson correlations between species occurrence and genetic distance to the focal species of different genera. **Figure S1.** Bayesian tree with posterior probabilities (a) and RAxML ML tree with bootstrap proportions from 1000 rapid bootstrap replicates (b) for *Tetranychus* species in China. **Figure S2.** Bayesian tree with posterior probabilities of 88 species. **Figure S3.** ML trees based on 1000 rapid bootstrap replicates of 88 species. **Figure S4.** Correlations between host specialization and local occurrence in the China dataset (a) and the global dataset (b).

## Data Availability

The phylogenetic trees generated during the current study are available in the Treebase: http://purl.org/phylo/treebase/phylows/study/TB2:S25372. Sequences can be retrieved from Genbank with accession numbers provided in Additional file 1: Table S2. The other data supporting findings are contained within the manuscript and in the supplemental files. If any additional information is necessary, please contact the corresponding author.

## Abbreviations

AF: Average frequency of occurrence
BI: Bayesian inference
DC: Number of distributed countries
HF: Number of host families
ML: Maximum likelihood
NOC: Total number of occurrences
PGLS: Phylogenetic generalized linear models
PIC: Phylogenetically independent contrasts function
