# Supplementary material for: Phylogenetic signals in pest abundance and distribution range of spider mites
Source: BMC Evol Biol. 2019 Dec 5;19:223. doi: 10.1186/s12862-019-1548-3 (PMC6896397; doi:10.1186/s12862-019-1548-3)

**Additional file 1**

**Phylogenetic signals in pest abundance and distribution range of spider mites**

Peng-Yu Jin^1†^, Jing-Tao Sun^1†^, Ary Hoffmann^2^, Yan-Fei Guo^1^, Jin-Cheng Zhou^3^, Yu-Xi Zhu^1^, Lei Chen^1^, and Xiao-Yue Hong^1*^

^*^ Correspondence: xyhong@njau.edu.cn

^1^ Department of Entomology, Nanjing Agricultural University, Nanjing, Jiangsu 210095, China

^2^ School of BioSciences, Bio21 Institute, The University of Melbourne, Melbourne, Victoria, Australia

^3^ School of Plant Protection, Shenyang Agricultural University, Shenyang, Liaoning, 110866, China

^†^These authors contributed equally to this work

**Table S1** Sample information for samples from China used in this study

| Pop ID | Locality | Sites | Host | Latitude | Longitude | time | Tur | Tug | Ttr | Tpu | Tpi | Tph | Tma | T lu | Tka | Tev | Pci | Avi |
| --- | --- | --- | --- | --- | --- | --- | --- | --- | --- | --- | --- | --- | --- | --- | --- | --- | --- | --- |
| 1 | Achen | Site1 | *Glycine max* | 45.55 | 126.99 | 2015 | 0 | 0 | 13 | 10 | 0 | 0 | 0 | 0 | 0 | 0 | 0 | 0 |
| 2 |  |  | *Phaseolus vulgaris* | 45.55 | 126.99 | 2015 | 0 | 0 | 1 | 19 | 0 | 0 | 0 | 0 | 0 | 0 | 0 | 0 |
| 3 |  |  | *Solanum melongena* | 45.55 | 126.99 | 2015 | 0 | 0 | 12 | 0 | 0 | 0 | 0 | 0 | 0 | 0 | 0 | 0 |
| 4 | Anqing | Site1 | *Gossypium spp* | 30.56 | 117.12 | 2008 | 0 | 0 | 0 | 0 | 0 | 0 | 0 | 0 | 58 | 0 | 0 | 0 |
| 5 |  | Site2 | *Prunus davidiana* | 30.49 | 117.04 | 2012 | 0 | 0 | 0 | 0 | 0 | 0 | 0 | 0 | 0 | 0 | 0 | 32 |
| 6 | Baise | Site1 | *Chaenomeles sinensis* | 23.87 | 106.64 | 2017 | 0 | 0 | 0 | 0 | 0 | 0 | 0 | 0 | 40 | 0 | 0 | 0 |
| 7 |  |  | *Ipomoea batatas* | 23.87 | 106.64 | 2017 | 0 | 0 | 0 | 0 | 10 | 0 | 0 | 0 | 0 | 0 | 0 | 0 |
| 8 |  |  | *Solanum melongena* | 23.87 | 106.64 | 2017 | 0 | 0 | 0 | 4 | 0 | 0 | 0 | 0 | 0 | 11 | 0 | 0 |
| 9 |  |  | *Vigna unguiculata* | 23.87 | 106.64 | 2017 | 0 | 0 | 0 | 30 | 0 | 0 | 0 | 0 | 0 | 0 | 0 | 0 |
| 10 | Baoding | Site1 | *Glycine max* | 38.55 | 115.28 | 2016 | 0 | 0 | 8 | 0 | 0 | 0 | 0 | 0 | 0 | 0 | 0 | 0 |
| 11 | Baoji | Site1 | *Phaseolus vulgaris* | 34.09 | 107.65 | 2014 | 0 | 0 | 60 | 0 | 0 | 0 | 0 | 0 | 0 | 0 | 0 | 0 |
| 12 | Baotou | Site1 | *Glycine max* | 40.56 | 109.84 | 2011 | 0 | 0 | 51 | 0 | 0 | 0 | 0 | 0 | 0 | 0 | 0 | 0 |
| 13 |  |  | *Pharbitis nil* | 40.56 | 109.84 | 2011 | 0 | 0 | 49 | 0 | 0 | 0 | 0 | 0 | 0 | 0 | 0 | 0 |
| 14 | Batang | Site1 | *Phaseolus vulgaris* | 30.01 | 99.11 | 2014 | 2 | 0 | 0 | 28 | 0 | 0 | 0 | 0 | 0 | 0 | 0 | 0 |
| 15 | Beibei | Site1 | *Solanum melongena* | 29.76 | 106.38 | 2013 | 0 | 0 | 0 | 3 | 0 | 0 | 0 | 0 | 0 | 0 | 0 | 0 |
| 16 |  |  | *Vigna unguiculata* | 29.76 | 106.38 | 2013 | 0 | 0 | 0 | 3 | 0 | 0 | 0 | 0 | 0 | 0 | 0 | 0 |
| 17 | Beidaihe | Site1 | *Benincasa Savi* | 39.83 | 119.48 | 2014 | 17 | 0 | 9 | 0 | 0 | 0 | 0 | 0 | 0 | 0 | 0 | 0 |
| 18 | Bengbu | Site1 | *Prunus salicina* | 32.88 | 117.36 | 2012 | 0 | 0 | 0 | 0 | 0 | 0 | 0 | 0 | 0 | 0 | 0 | 22 |
| 19 |  | Site2 | *Prunus salicina* | 32.87 | 117.28 | 2010 | 0 | 0 | 0 | 0 | 0 | 0 | 0 | 0 | 3 | 0 | 0 | 0 |
| 20 | Cangzhou | Site1 | *Cucurbita moschata* | 38.31 | 116.97 | 2014 | 0 | 0 | 3 | 0 | 0 | 0 | 0 | 0 | 0 | 0 | 0 | 0 |
| 21 |  |  | *Solanum lycopersicum* | 38.31 | 116.97 | 2014 | 0 | 0 | 3 | 0 | 0 | 0 | 0 | 0 | 0 | 0 | 0 | 0 |
| 22 |  |  | *Vigna radiata* | 38.31 | 116.97 | 2014 | 0 | 0 | 7 | 0 | 0 | 0 | 0 | 0 | 0 | 0 | 0 | 0 |
| 23 |  | Site2 | *Solanum melongena* | 38.3 | 116.76 | 2014 | 0 | 0 | 46 | 0 | 0 | 0 | 0 | 0 | 0 | 0 | 0 | 0 |
| 24 |  |  | *Trivhosantnes Kirilouii Manim* | 38.3 | 116.76 | 2014 | 0 | 0 | 21 | 0 | 0 | 0 | 0 | 0 | 0 | 0 | 0 | 0 |
| 25 |  | Site3 | *Amygdalus persicai* | 37.8 | 116.64 | 2016 | 0 | 0 | 0 | 0 | 0 | 0 | 0 | 0 | 0 | 0 | 0 | 3 |
| 26 |  |  | *Vigna radiata* | 37.8 | 116.64 | 2016 | 0 | 3 | 0 | 0 | 0 | 0 | 0 | 0 | 0 | 0 | 0 | 0 |
| 27 | Changchun | Site1 | *Armeniaca vulgaris* | 43.85 | 125.31 | 2011 | 0 | 0 | 0 | 0 | 0 | 0 | 0 | 0 | 0 | 0 | 0 | 40 |
| 28 |  | Site2 | *Zea mays* | 43.59 | 125.64 | 2014 | 0 | 0 | 0 | 30 | 0 | 0 | 0 | 0 | 0 | 0 | 0 | 0 |
| 29 |  | Site3 | *Glycine max* | 43.57 | 125.68 | 2014 | 0 | 0 | 0 | 12 | 0 | 0 | 0 | 0 | 0 | 0 | 0 | 0 |
| 30 |  |  | *Solanum melongena* | 43.57 | 125.68 | 2014 | 4 | 0 | 94 | 5 | 0 | 0 | 0 | 0 | 0 | 0 | 0 | 0 |
| 31 |  |  | *Vigna unguiculata* | 43.57 | 125.68 | 2014 | 0 | 0 | 48 | 1 | 0 | 0 | 0 | 0 | 0 | 0 | 0 | 0 |
| 32 |  | Site4 | *Cucumis sativus* | 43.56 | 125.67 | 2014 | 0 | 0 | 20 | 0 | 0 | 0 | 0 | 0 | 0 | 0 | 0 | 0 |
| 33 |  |  | *Portulaca oleracea* | 43.56 | 125.67 | 2014 | 0 | 0 | 5 | 45 | 0 | 0 | 0 | 0 | 0 | 0 | 0 | 0 |
| 34 |  |  | *Solanum melongena* | 43.56 | 125.67 | 2014 | 0 | 0 | 90 | 9 | 0 | 0 | 0 | 0 | 0 | 0 | 0 | 0 |
| 35 | Changle | Site1 | *Gossypium hirsutum* | 36.7 | 118.81 | 2011 | 0 | 0 | 3 | 0 | 0 | 0 | 0 | 0 | 0 | 0 | 0 | 0 |
| 36 | Changli | Site1 | *Malus domestica* | 39.71 | 119.21 | 2008 | 0 | 44 | 0 | 0 | 0 | 0 | 0 | 0 | 0 | 0 | 0 | 0 |
| 37 |  | Site2 | *Phaseolus vulgaris* | 39.71 | 119.21 | 2011 | 0 | 0 | 56 | 0 | 0 | 0 | 0 | 0 | 0 | 0 | 0 | 0 |
| 38 | Changping | Site1 | *Malus pumila* | 41.23 | 116.06 | 2011 | 0 | 0 | 0 | 0 | 0 | 0 | 0 | 0 | 0 | 0 | 0 | 3 |
| 39 |  | Site2 | *Phaseolus vulgaris* | 40.23 | 116.06 | 2011 | 0 | 0 | 12 | 0 | 0 | 0 | 0 | 0 | 0 | 0 | 0 | 0 |
| 40 | Changsha | Site1 | *Solanum melongena* | 28.26 | 112.87 | 2011 | 0 | 0 | 40 | 0 | 0 | 0 | 0 | 0 | 0 | 0 | 0 | 0 |
| 41 |  | Site2 | *Vigna unguiculata* | 28.19 | 112.75 | 2008 | 0 | 0 | 0 | 3 | 0 | 0 | 0 | 0 | 0 | 0 | 0 | 0 |
| 42 |  | Site3 | *Prunus davidiana* | 28.12 | 112.99 | 2012 | 0 | 0 | 0 | 0 | 0 | 0 | 0 | 0 | 0 | 0 | 0 | 3 |
| 43 | Changyi | Site1 | *Gossypium hirsutum* | 36.86 | 119.42 | 2011 | 0 | 0 | 3 | 0 | 0 | 0 | 0 | 0 | 0 | 0 | 0 | 0 |
| 44 |  | Site2 | *Solanum lycopersicum* | 36.76 | 119.6 | 2014 | 0 | 0 | 40 | 1 | 0 | 0 | 0 | 0 | 0 | 0 | 0 | 39 |
| 45 |  |  | *Solanum melongena* | 36.76 | 119.6 | 2014 | 0 | 0 | 41 | 4 | 0 | 0 | 0 | 0 | 0 | 0 | 0 | 0 |
| 46 | Changzhi | Site1 | *Solanum melongena* | 35.92 | 113.57 | 2014 | 0 | 0 | 91 | 0 | 0 | 0 | 0 | 0 | 0 | 0 | 0 | 0 |
| 47 |  |  | *Vigna unguiculata* | 35.92 | 113.57 | 2014 | 0 | 0 | 67 | 0 | 0 | 0 | 0 | 0 | 0 | 0 | 0 | 0 |
| 48 | Chaozhou | Site1 | *Citrus reticulata Blanco* | 23.65 | 116.62 | 2015 | 0 | 0 | 0 | 0 | 0 | 0 | 0 | 0 | 0 | 0 | 44 | 0 |
| 49 | Chengde | Site1 | *Glycine max* | 40.94 | 117.94 | 2010 | 0 | 0 | 0 | 0 | 0 | 40 | 0 | 0 | 0 | 0 | 0 | 0 |
| 50 | Chengdu | Site1 | *Cucumis sativus* | 30.71 | 103.86 | 2016 | 0 | 0 | 0 | 0 | 0 | 0 | 0 | 0 | 0 | 30 | 0 | 0 |
| 51 |  |  | *Lycopersicon esculentum* | 30.71 | 103.86 | 2016 | 0 | 0 | 5 | 0 | 0 | 0 | 0 | 0 | 0 | 0 | 0 | 0 |
| 52 |  |  | *Solanum lycopersicum* | 30.71 | 103.86 | 2016 | 0 | 0 | 14 | 0 | 0 | 0 | 0 | 0 | 0 | 0 | 0 | 0 |
| 53 |  |  | *Solanum melongena* | 30.71 | 103.86 | 2016 | 0 | 0 | 16 | 0 | 0 | 0 | 0 | 0 | 0 | 0 | 0 | 0 |
| 54 |  |  | *Zea mays* | 30.71 | 103.86 | 2016 | 0 | 0 | 12 | 0 | 0 | 0 | 0 | 0 | 0 | 0 | 0 | 0 |
| 55 |  | Site2 | *Phaseolus vulgaris* | 30.6 | 104.25 | 2014 | 0 | 0 | 4 | 49 | 0 | 2 | 0 | 0 | 0 | 0 | 0 | 0 |
| 56 |  |  | *Vigna unguiculata* | 30.6 | 104.25 | 2014 | 0 | 0 | 0 | 3 | 0 | 0 | 0 | 0 | 0 | 0 | 0 | 0 |
| 57 |  | Site3 | *Solanum melongena* | 30.52 | 103.95 | 2013 | 0 | 0 | 3 | 0 | 0 | 0 | 0 | 0 | 0 | 0 | 0 | 0 |
| 58 | Chengzhou | Site1 | *Argyreia seguini* | 25.76 | 112.97 | 2017 | 0 | 0 | 0 | 0 | 3 | 0 | 0 | 0 | 6 | 0 | 0 | 0 |
| 59 |  |  | *Castanea mollissima* | 25.76 | 112.97 | 2017 | 0 | 0 | 0 | 0 | 0 | 0 | 0 | 0 | 6 | 0 | 0 | 0 |
| 60 |  |  | *Euphorbia hirta* | 25.76 | 112.97 | 2017 | 0 | 0 | 0 | 0 | 0 | 0 | 0 | 0 | 5 | 0 | 0 | 0 |
| 61 |  |  | *Solanum melongena* | 25.76 | 112.97 | 2017 | 0 | 0 | 0 | 0 | 0 | 0 | 0 | 0 | 6 | 0 | 0 | 0 |
| 62 |  | Site2 | *Colocasia esculenta* | 25.74 | 113 | 2017 | 0 | 0 | 0 | 0 | 0 | 0 | 0 | 0 | 21 | 0 | 0 | 0 |
| 63 |  |  | *Ipomoea aquatica* | 25.74 | 113 | 2017 | 0 | 0 | 0 | 0 | 4 | 0 | 0 | 0 | 0 | 0 | 0 | 0 |
| 64 |  |  | *Ipomoea batatas* | 25.74 | 113 | 2017 | 0 | 0 | 0 | 0 | 6 | 0 | 0 | 0 | 0 | 0 | 0 | 0 |
| 65 |  |  | *Pyrus spp* | 25.74 | 113 | 2017 | 0 | 0 | 0 | 0 | 0 | 0 | 0 | 0 | 30 | 0 | 0 | 0 |
| 66 |  |  | *Solanum melongena* | 25.74 | 113 | 2017 | 0 | 0 | 0 | 0 | 0 | 0 | 0 | 0 | 4 | 0 | 0 | 0 |
| 67 |  |  | *Vigna unguiculata* | 25.74 | 113 | 2017 | 0 | 0 | 0 | 0 | 3 | 0 | 0 | 0 | 8 | 0 | 0 | 0 |
| 68 | Chongqing | Site1 | *Basella alba* | 29.82 | 106.42 | 2013 | 0 | 0 | 3 | 0 | 0 | 0 | 0 | 0 | 0 | 0 | 0 | 0 |
| 69 |  |  | *Cucumis sativus* | 29.82 | 106.42 | 2013 | 0 | 0 | 0 | 3 | 0 | 0 | 0 | 0 | 0 | 0 | 0 | 0 |
| 70 |  |  | *Solanum melongena* | 29.82 | 106.42 | 2013 | 0 | 0 | 0 | 3 | 0 | 0 | 0 | 0 | 0 | 0 | 0 | 0 |
| 71 | Cixi | Site1 | *Benincasa hispida* | 30.31 | 120.44 | 2016 | 0 | 0 | 0 | 0 | 0 | 0 | 0 | 0 | 5 | 0 | 0 | 0 |
| 72 |  |  | *Colocasia esculentam* | 30.31 | 120.44 | 2016 | 0 | 0 | 0 | 0 | 0 | 0 | 0 | 0 | 20 | 0 | 0 | 0 |
| 73 |  |  | *Cucurbita moschata* | 30.31 | 120.44 | 2016 | 0 | 0 | 0 | 0 | 0 | 0 | 0 | 0 | 3 | 0 | 0 | 0 |
| 74 |  |  | *Solanum melongena* | 30.31 | 120.44 | 2016 | 0 | 0 | 0 | 0 | 0 | 0 | 0 | 0 | 37 | 0 | 0 | 0 |
| 75 |  | Site2 | *Gossypium hirsutum* | 30.23 | 121.3 | 2012 | 0 | 0 | 0 | 0 | 0 | 0 | 0 | 0 | 3 | 0 | 0 | 0 |
| 76 |  | Site3 | *Gossypium spp* | 30.19 | 121.35 | 2008 | 0 | 0 | 0 | 0 | 0 | 0 | 0 | 0 | 3 | 0 | 0 | 0 |
| 77 |  | Site4 | *Gossypium herbaceum* | 30.16 | 121.26 | 2011 | 0 | 0 | 40 | 0 | 0 | 0 | 0 | 0 | 0 | 0 | 0 | 0 |
| 78 |  |  | *Gossypium hirsutum* | 30.16 | 121.26 | 2011 | 0 | 0 | 0 | 50 | 0 | 3 | 0 | 0 | 0 | 0 | 0 | 0 |
| 79 |  |  | *Prunus davidiana* | 30.16 | 121.26 | 2011 | 0 | 0 | 0 | 0 | 0 | 0 | 0 | 0 | 0 | 0 | 0 | 24 |
| 80 | Fugong | Site1 | *Cucumis sativus* | 26.56 | 98.91 | 2013 | 0 | 0 | 0 | 50 | 0 | 0 | 0 | 0 | 0 | 0 | 0 | 0 |
| 81 | Gaglazy | Site1 | *Solanum melongena* | 50.4 | 124.07 | 2008 | 0 | 0 | 3 | 0 | 0 | 0 | 0 | 0 | 0 | 0 | 0 | 0 |
| 82 | Guangzhou | Site1 | *Amaranthus tricolor* | 23.18 | 113.23 | 2015 | 0 | 0 | 0 | 0 | 0 | 0 | 0 | 22 | 0 | 0 | 0 | 0 |
| 83 |  |  | *Benincasa Savi* | 23.18 | 113.23 | 2015 | 0 | 0 | 3 | 0 | 0 | 0 | 0 | 0 | 0 | 0 | 0 | 0 |
| 84 |  |  | *Ipomoea batatas* | 23.18 | 113.23 | 2015 | 0 | 0 | 13 | 0 | 0 | 0 | 0 | 0 | 0 | 0 | 0 | 0 |
| 85 |  |  | *Luffa cylindrica* | 23.18 | 113.23 | 2015 | 0 | 0 | 3 | 0 | 0 | 0 | 0 | 0 | 0 | 0 | 0 | 0 |
| 86 |  |  | *Phaseolus vulgaris* | 23.18 | 113.23 | 2015 | 0 | 0 | 41 | 0 | 0 | 0 | 0 | 0 | 0 | 0 | 0 | 0 |
| 87 |  |  | *Solanum melongena* | 23.18 | 113.23 | 2015 | 0 | 0 | 3 | 0 | 0 | 0 | 0 | 0 | 0 | 0 | 0 | 0 |
| 88 |  | Site2 | *Benincasa Savi* | 23.16 | 113.35 | 2013 | 0 | 0 | 30 | 0 | 0 | 0 | 0 | 0 | 0 | 0 | 0 | 0 |
| 89 |  |  | *Daucus carota* | 23.16 | 113.35 | 2013 | 0 | 0 | 0 | 0 | 0 | 0 | 0 | 4 | 0 | 0 | 0 | 0 |
| 90 |  |  | *Ipomoea batatas* | 23.16 | 113.35 | 2013 | 0 | 0 | 0 | 0 | 3 | 0 | 0 | 0 | 0 | 0 | 0 | 0 |
| 91 |  | Site3 | *Ipomoea batatas* | 23.09 | 113.3 | 2017 | 0 | 0 | 0 | 0 | 3 | 0 | 0 | 0 | 0 | 0 | 0 | 0 |
| 92 |  |  | *Solanum melongena* | 23.09 | 113.3 | 2017 | 0 | 0 | 0 | 0 | 0 | 0 | 0 | 0 | 0 | 3 | 0 | 0 |
| 93 |  | Site4 | *Pseudocydonia sinensism* | 22.87 | 113.35 | 2011 | 3 | 0 | 0 | 0 | 0 | 0 | 0 | 0 | 0 | 0 | 0 | 0 |
| 94 | Guilin | Site1 | *Abelmoschus esculentus* | 25.27 | 110.25 | 2017 | 0 | 0 | 0 | 21 | 0 | 0 | 0 | 0 | 0 | 0 | 0 | 0 |
| 95 |  |  | *Glycine max* | 25.27 | 110.25 | 2017 | 0 | 0 | 0 | 10 | 0 | 0 | 0 | 0 | 0 | 0 | 0 | 0 |
| 96 |  |  | *Ipomoea batatas* | 25.27 | 110.25 | 2017 | 0 | 0 | 0 | 6 | 0 | 0 | 0 | 0 | 0 | 0 | 0 | 0 |
| 97 |  |  | *Luffa cylindrica* | 25.27 | 110.25 | 2017 | 0 | 0 | 0 | 33 | 0 | 0 | 0 | 0 | 0 | 0 | 0 | 0 |
| 98 |  |  | *Solanum melongena* | 25.27 | 110.25 | 2017 | 0 | 0 | 0 | 30 | 0 | 0 | 0 | 0 | 0 | 0 | 0 | 0 |
| 99 |  |  | *Vigna unguiculata* | 25.27 | 110.25 | 2017 | 0 | 0 | 0 | 3 | 0 | 0 | 0 | 0 | 4 | 0 | 0 | 0 |
| 100 | Guiyang | Site1 | *Solanum lycopersicum* | 26.42 | 106.66 | 2014 | 66 | 0 | 0 | 0 | 0 | 0 | 0 | 0 | 0 | 0 | 0 | 0 |
| 101 | Haba | Site1 | *Cucumis sativus* | 27.38 | 100.14 | 2013 | 0 | 0 | 0 | 50 | 0 | 0 | 0 | 0 | 0 | 0 | 0 | 0 |
| 102 |  |  | *Solanum melongena* | 27.38 | 100.14 | 2013 | 0 | 0 | 0 | 74 | 0 | 0 | 0 | 0 | 0 | 0 | 0 | 0 |
| 103 | Haerbin | Site1 | *Cucumis sativus* | 46.42 | 125.99 | 2014 | 0 | 0 | 29 | 0 | 0 | 0 | 0 | 0 | 0 | 0 | 0 | 0 |
| 104 |  |  | *Solanum lycopersicum* | 46.42 | 125.99 | 2014 | 0 | 0 | 0 | 42 | 0 | 0 | 0 | 0 | 0 | 0 | 0 | 0 |
| 105 |  |  | *Solanum melongena* | 46.42 | 125.99 | 2014 | 0 | 0 | 0 | 34 | 0 | 0 | 0 | 0 | 0 | 0 | 0 | 0 |
| 106 |  | Site2 | *Vigna unguiculata* | 46.33 | 125.99 | 2014 | 0 | 0 | 3 | 0 | 0 | 0 | 0 | 0 | 0 | 0 | 0 | 0 |
| 107 |  |  | *Zea mays* | 46.33 | 125.99 | 2014 | 0 | 0 | 7 | 0 | 0 | 0 | 0 | 0 | 0 | 0 | 0 | 0 |
| 108 |  | Site3 | *Citrullus lanatus* | 45.83 | 126.79 | 2010 | 0 | 19 | 0 | 0 | 0 | 0 | 0 | 0 | 0 | 0 | 0 | 0 |
| 109 |  | Site4 | *Phaseolus vulgaris* | 45.83 | 126.46 | 2009 | 0 | 0 | 3 | 0 | 0 | 0 | 0 | 0 | 0 | 0 | 0 | 0 |
| 110 |  | Site5 | *Glycine max* | 45.82 | 126.56 | 2011 | 0 | 0 | 40 | 0 | 0 | 0 | 0 | 0 | 0 | 0 | 0 | 0 |
| 111 |  | Site6 | *Zea mays* | 45.42 | 126.43 | 2016 | 0 | 0 | 3 | 0 | 0 | 0 | 0 | 0 | 0 | 0 | 0 | 0 |
| 112 |  | Site7 | *Phaseolus vulgaris* | 45.34 | 126.59 | 2015 | 0 | 0 | 0 | 3 | 0 | 0 | 0 | 0 | 0 | 0 | 0 | 0 |
| 113 |  |  | *Solanum lycopersicum* | 45.34 | 126.59 | 2015 | 0 | 0 | 20 | 0 | 0 | 0 | 0 | 0 | 0 | 0 | 0 | 0 |
| 114 |  |  | *Solanum melongena* | 45.34 | 126.59 | 2015 | 0 | 0 | 30 | 0 | 0 | 0 | 0 | 0 | 0 | 0 | 0 | 0 |
| 115 |  |  | *Vigna unguiculata* | 45.34 | 126.59 | 2015 | 7 | 0 | 0 | 0 | 0 | 0 | 0 | 0 | 0 | 0 | 0 | 0 |
| 116 | Handan | Site1 | *Solanum melongena* | 36.84 | 114.59 | 2014 | 0 | 0 | 23 | 0 | 0 | 0 | 0 | 0 | 0 | 0 | 0 | 0 |
| 117 |  | Site2 | *Gossypium hirsutum* | 36.64 | 114.66 | 2012 | 0 | 0 | 35 | 0 | 0 | 0 | 0 | 0 | 0 | 0 | 0 | 0 |
| 118 | Hangzhou | Site1 | *Vigna unguiculata* | 30.32 | 120.05 | 2014 | 0 | 0 | 0 | 0 | 0 | 0 | 0 | 0 | 9 | 0 | 0 | 0 |
| 119 | Hebeinongda | Site1 | *Cucumis sativus* | 38.86 | 115.49 | 2017 | 0 | 0 | 3 | 0 | 0 | 0 | 0 | 0 | 0 | 0 | 0 | 0 |
| 120 |  |  | *Vigna unguiculata* | 38.86 | 115.49 | 2017 | 0 | 0 | 3 | 0 | 0 | 0 | 0 | 0 | 0 | 0 | 0 | 0 |
| 121 | Hohhot | Site1 | *Cucurbita moschata* | 40.79 | 111.79 | 2014 | 0 | 0 | 61 | 0 | 0 | 0 | 0 | 0 | 0 | 0 | 0 | 0 |
| 122 |  |  | *Solanum lycopersicum* | 40.79 | 111.79 | 2014 | 0 | 0 | 72 | 0 | 0 | 0 | 0 | 0 | 0 | 0 | 0 | 0 |
| 123 |  |  | *Solanum melongena* | 40.79 | 111.79 | 2014 | 0 | 0 | 72 | 0 | 0 | 0 | 0 | 0 | 0 | 0 | 0 | 0 |
| 124 |  | Site2 | *Portulaca oleracea* | 40.79 | 111.78 | 2014 | 0 | 0 | 10 | 0 | 0 | 0 | 0 | 0 | 0 | 0 | 0 | 0 |
| 125 |  |  | *Solanum melongena* | 40.79 | 111.78 | 2014 | 0 | 0 | 61 | 0 | 0 | 0 | 0 | 0 | 0 | 0 | 0 | 0 |
| 126 | Huaian | Site1 | *Armeniaca vulgaris* | 40.6 | 113.69 | 2017 | 2 | 0 | 1 | 0 | 0 | 0 | 0 | 0 | 0 | 0 | 0 | 0 |
| 127 |  |  | *Cucumis sativus* | 40.6 | 113.69 | 2017 | 0 | 0 | 3 | 0 | 0 | 0 | 0 | 0 | 0 | 0 | 0 | 0 |
| 128 | Huizhou | Site1 | *Amaranthus tricolor* | 22.83 | 114.81 | 2016 | 0 | 0 | 51 | 0 | 0 | 0 | 0 | 0 | 0 | 0 | 0 | 0 |
| 129 |  |  | *Colocasia esculenta* | 22.83 | 114.81 | 2016 | 0 | 0 | 0 | 0 | 0 | 0 | 0 | 0 | 36 | 0 | 0 | 0 |
| 130 |  |  | *Ipomoea aquatica* | 22.83 | 114.81 | 2016 | 0 | 0 | 3 | 0 | 0 | 0 | 0 | 0 | 0 | 0 | 0 | 0 |
| 131 |  |  | *Solanum melongena* | 22.83 | 114.81 | 2016 | 0 | 0 | 37 | 0 | 0 | 0 | 0 | 0 | 0 | 0 | 0 | 0 |
| 132 | Hutiaoxia | Site1 | *Cucumis sativus* | 27.37 | 100.15 | 2013 | 0 | 0 | 0 | 3 | 0 | 0 | 0 | 0 | 0 | 0 | 0 | 0 |
| 133 |  |  | *Vigna unguiculata* | 27.37 | 100.15 | 2013 | 0 | 0 | 0 | 31 | 0 | 0 | 0 | 0 | 0 | 0 | 0 | 0 |
| 134 | Jeminay | Site1 | *Cucumis sativus* | 47.46 | 85.92 | 2011 | 0 | 0 | 82 | 0 | 0 | 0 | 0 | 0 | 0 | 0 | 0 | 0 |
| 135 | Jinan | Site1 | *Glycine max* | 36.69 | 116.94 | 2011 | 0 | 0 | 3 | 0 | 0 | 0 | 0 | 0 | 0 | 0 | 0 | 0 |
| 136 |  |  | *Gossypium hirsutum* | 36.69 | 116.94 | 2011 | 0 | 0 | 3 | 0 | 0 | 0 | 0 | 0 | 0 | 0 | 0 | 0 |
| 137 |  | Site2 | *Gossypium spp* | 36.63 | 116.87 | 2008 | 0 | 0 | 7 | 0 | 0 | 0 | 0 | 0 | 0 | 0 | 0 | 0 |
| 138 |  |  | *Malus domestica* | 36.63 | 116.87 | 2008 | 0 | 39 | 0 | 0 | 0 | 0 | 0 | 0 | 0 | 0 | 0 | 0 |
| 139 | Jingning | Site1 | *Zea mays* | 35.53 | 105.73 | 2011 | 3 | 0 | 0 | 0 | 0 | 0 | 0 | 0 | 0 | 0 | 0 | 0 |
| 140 |  | Site2 | *Malus spp.* | 35.52 | 105.71 | 2010 | 0 | 0 | 0 | 3 | 0 | 0 | 0 | 0 | 0 | 0 | 0 | 0 |
| 141 |  |  | *Zea mays* | 35.52 | 105.71 | 2010 | 0 | 40 | 0 | 0 | 0 | 0 | 0 | 0 | 0 | 0 | 0 | 0 |
| 142 | Jingzhou | Site1 | *Vigna unguiculata* | 30.37 | 112.32 | 2009 | 0 | 0 | 0 | 3 | 0 | 0 | 0 | 0 | 3 | 0 | 0 | 0 |
| 143 | Jiujiang | Site1 | *Phaseolus vulgaris* | 29.7 | 116.06 | 2010 | 3 | 0 | 0 | 0 | 0 | 0 | 0 | 0 | 0 | 0 | 0 | 0 |
| 144 |  | Site2 | *Gossypium herbaceum* | 29.7 | 116.05 | 2008 | 0 | 0 | 0 | 7 | 0 | 0 | 0 | 0 | 0 | 0 | 0 | 0 |
| 145 |  |  | *Gossypium spp* | 29.7 | 116.05 | 2008 | 49 | 0 | 0 | 0 | 0 | 0 | 0 | 0 | 0 | 0 | 0 | 0 |
| 146 | Jiuquan | Site1 | *Solanum melongena* | 39.71 | 98.44 | 2010 | 0 | 0 | 61 | 0 | 0 | 0 | 0 | 0 | 0 | 0 | 0 | 0 |
| 147 |  | Site2 | *Solanum melongena* | 39.71 | 98.48 | 2011 | 0 | 0 | 0 | 20 | 0 | 0 | 0 | 0 | 0 | 0 | 0 | 0 |
| 148 | Kunming | Site1 | *Solanum lycopersicum* | 25.12 | 102.75 | 2013 | 26 | 0 | 0 | 0 | 0 | 0 | 0 | 0 | 0 | 0 | 0 | 0 |
| 149 | Lanzhou | Site1 | *Trifolium repens* | 36.01 | 103.77 | 2009 | 0 | 0 | 1 | 0 | 0 | 0 | 0 | 0 | 34 | 0 | 0 | 0 |
| 150 | Leizhou | Site1 | *Ipomoea batatas* | 21.29 | 110.35 | 2013 | 0 | 0 | 0 | 0 | 0 | 0 | 0 | 3 | 0 | 0 | 0 | 0 |
| 151 |  | Site1 | *Ipomoea batatas* | 20.91 | 110.11 | 2013 | 0 | 0 | 0 | 0 | 12 | 0 | 0 | 0 | 3 | 0 | 0 | 0 |
| 152 |  |  | *Vigna unguiculata* | 20.91 | 110.11 | 2013 | 0 | 0 | 3 | 0 | 2 | 0 | 0 | 0 | 0 | 0 | 0 | 0 |
| 153 | Lingshui | Site1 | *Glycine max* | 18.52 | 109.95 | 2013 | 0 | 0 | 0 | 0 | 0 | 0 | 45 | 0 | 0 | 0 | 0 | 0 |
| 154 | Linxia | Site1 | *Glycine max* | 35.36 | 103.71 | 2016 | 0 | 0 | 3 | 0 | 0 | 0 | 0 | 0 | 0 | 0 | 0 | 0 |
| 155 |  |  | *Phaseolus vulgaris* | 35.36 | 103.71 | 2016 | 0 | 0 | 8 | 0 | 0 | 0 | 0 | 0 | 0 | 0 | 0 | 0 |
| 156 |  |  | *Solanum melongena* | 35.36 | 103.71 | 2016 | 0 | 0 | 4 | 9 | 0 | 0 | 0 | 0 | 0 | 0 | 0 | 0 |
| 157 |  |  | *Zea mays* | 35.36 | 103.71 | 2016 | 0 | 0 | 3 | 2 | 0 | 0 | 0 | 0 | 0 | 0 | 0 | 0 |
| 158 | Lixian | Site1 | *Amygdalus persicas* | 34.31 | 105.56 | 2016 | 0 | 0 | 0 | 3 | 0 | 0 | 0 | 0 | 0 | 0 | 0 | 0 |
| 159 |  |  | *Malus pumila* | 34.31 | 105.56 | 2016 | 0 | 0 | 0 | 0 | 0 | 0 | 0 | 0 | 0 | 0 | 0 | 10 |
| 160 |  |  | *Phaseolus vulgaris* | 34.31 | 105.56 | 2016 | 0 | 0 | 8 | 0 | 0 | 0 | 0 | 0 | 0 | 0 | 0 | 0 |
| 161 | Longyan | Site1 | *Glycine max* | 25.1 | 116.99 | 2010 | 0 | 0 | 40 | 0 | 0 | 0 | 0 | 0 | 0 | 0 | 0 | 0 |
| 162 | Mayang | Site1 | *Phaseolus vulgaris* | 27.86 | 109.8 | 2014 | 0 | 0 | 0 | 0 | 33 | 0 | 0 | 0 | 0 | 0 | 0 | 0 |
| 163 | Meishan | Site1 | *Gossypium herbaceum* | 30.08 | 103.86 | 2008 | 0 | 0 | 0 | 0 | 0 | 0 | 0 | 0 | 36 | 0 | 0 | 0 |
| 164 |  |  | *Gossypium spp* | 30.08 | 103.86 | 2008 | 0 | 0 | 0 | 9 | 0 | 0 | 0 | 0 | 0 | 0 | 0 | 0 |
| 165 |  | Site2 | *Citrus reticulata Blanco* | 30.07 | 103.88 | 2016 | 0 | 0 | 0 | 0 | 0 | 0 | 0 | 0 | 0 | 0 | 10 | 0 |
| 166 |  | Site3 | *Citrus reticulata Blanco* | 30.04 | 103.84 | 2014 | 0 | 0 | 0 | 0 | 0 | 0 | 0 | 0 | 0 | 0 | 87 | 0 |
| 167 | Mudanjiang | Site1 | *Phaseolus vulgaris* | 44.55 | 129.58 | 2009 | 0 | 0 | 3 | 0 | 0 | 0 | 0 | 0 | 0 | 0 | 0 | 0 |
| 168 |  |  | *Phaseolus vulgaris* | 44.55 | 129.58 | 2009 | 0 | 0 | 0 | 0 | 0 | 0 | 0 | 0 | 8 | 0 | 0 | 0 |
| 169 | Nanchang | Site1 | *Cucurbita moschata* | 28.77 | 115.83 | 2016 | 0 | 0 | 0 | 63 | 0 | 0 | 0 | 0 | 0 | 0 | 0 | 0 |
| 170 |  |  | *Ipomoea aquatica* | 28.77 | 115.83 | 2016 | 0 | 0 | 0 | 0 | 0 | 0 | 0 | 0 | 32 | 0 | 0 | 0 |
| 171 |  |  | *Phaseolus vulgaris* | 28.77 | 115.83 | 2016 | 0 | 0 | 0 | 13 | 0 | 0 | 0 | 0 | 0 | 0 | 0 | 0 |
| 172 |  |  | *Zea mays* | 28.77 | 115.83 | 2016 | 0 | 0 | 15 | 8 | 0 | 0 | 0 | 0 | 0 | 0 | 0 | 0 |
| 173 | Nanhui | Site1 | *Gossypium spp* | 31.06 | 121.74 | 2008 | 0 | 0 | 0 | 0 | 0 | 0 | 0 | 0 | 11 | 0 | 0 | 0 |
| 174 | Nanjing | Site1 | *Prunus davidiana* | 32.08 | 118.85 | 2011 | 0 | 0 | 0 | 0 | 0 | 0 | 0 | 0 | 0 | 0 | 0 | 32 |
| 175 |  |  | *Solanum melongena* | 32.08 | 118.85 | 2011 | 0 | 0 | 24 | 0 | 0 | 0 | 0 | 0 | 0 | 0 | 0 | 0 |
| 176 |  | Site2 | *Glycine max* | 32.03 | 118.63 | 2014 | 0 | 0 | 3 | 0 | 0 | 0 | 0 | 0 | 0 | 0 | 0 | 0 |
| 177 |  |  | *Vigna unguiculata* | 32.03 | 118.63 | 2014 | 0 | 0 | 9 | 0 | 0 | 0 | 0 | 0 | 0 | 0 | 0 | 0 |
| 178 | Nanning | Site1 | *Citrus reticulata Blanco* | 22.82 | 108.37 | 2015 | 0 | 0 | 0 | 0 | 0 | 0 | 0 | 0 | 0 | 0 | 36 | 0 |
| 179 | Nanping | Site1 | *Solanum melongena* | 26.67 | 118.15 | 2011 | 0 | 0 | 0 | 0 | 3 | 0 | 0 | 0 | 0 | 0 | 0 | 0 |
| 180 | Pengze | Site1 | *Gossypium hirsutum* | 29.88 | 116.66 | 2012 | 0 | 0 | 0 | 0 | 0 | 0 | 0 | 0 | 3 | 0 | 0 | 0 |
| 181 | Pingliang | Site1 | *Malus pumila* | 35.57 | 106.63 | 2016 | 0 | 0 | 0 | 3 | 0 | 0 | 0 | 0 | 0 | 0 | 0 | 0 |
| 182 | Pingyin | Site1 | *Gossypium hirsutum* | 36.29 | 116.4 | 2012 | 0 | 0 | 68 | 0 | 0 | 0 | 0 | 0 | 0 | 0 | 0 | 0 |
| 183 |  | Site2 | *Glycine max* | 36.26 | 116.58 | 2010 | 0 | 0 | 59 | 0 | 0 | 0 | 0 | 0 | 0 | 0 | 0 | 0 |
| 184 | Qingdao | Site1 | *Prunus davidiana* | 36.09 | 120.41 | 2011 | 0 | 0 | 0 | 0 | 0 | 0 | 0 | 0 | 0 | 0 | 0 | 24 |
| 185 | Qinhuangdao | Site1 | *Solanum lycopersicum* | 39.83 | 119.49 | 2014 | 26 | 0 | 2 | 0 | 0 | 0 | 0 | 0 | 0 | 0 | 0 | 0 |
| 186 |  |  | *Solanum melongena* | 39.83 | 119.49 | 2014 | 0 | 0 | 3 | 0 | 0 | 0 | 0 | 0 | 0 | 0 | 0 | 0 |
| 187 |  | Site2 | *Phaseolus vulgaris* | 39.83 | 119.46 | 2014 | 0 | 0 | 3 | 0 | 0 | 0 | 0 | 0 | 0 | 0 | 0 | 0 |
| 188 |  |  | *Solanum lycopersicum* | 39.83 | 119.46 | 2014 | 0 | 0 | 3 | 0 | 0 | 0 | 0 | 0 | 0 | 0 | 0 | 0 |
| 189 | Qiu county | Site1 | *Gossypium spp* | 36.86 | 115.2 | 2008 | 0 | 0 | 3 | 0 | 0 | 0 | 0 | 0 | 0 | 0 | 0 | 0 |
| 190 | Quanzhou | Site1 | *Citrus reticulata Blanco* | 25.35 | 118.26 | 2014 | 0 | 0 | 0 | 0 | 0 | 0 | 0 | 0 | 0 | 0 | 48 | 0 |
| 191 |  |  | *Phaseolus vulgaris* | 25.35 | 118.26 | 2014 | 0 | 0 | 0 | 0 | 0 | 0 | 0 | 0 | 36 | 0 | 0 | 0 |
| 192 |  |  | *Solanum melongena* | 25.35 | 118.26 | 2014 | 0 | 0 | 60 | 0 | 0 | 0 | 0 | 0 | 0 | 0 | 0 | 0 |
| 193 | Renxian | Site1 | *Cucurbita moschata* | 37.14 | 114.64 | 2010 | 0 | 0 | 0 | 0 | 0 | 0 | 0 | 0 | 4 | 0 | 0 | 0 |
| 194 |  |  | *Gossypium hirsutum* | 37.14 | 114.64 | 2012 | 0 | 0 | 48 | 0 | 0 | 0 | 0 | 0 | 0 | 0 | 0 | 0 |
| 195 | Rudong | Site1 | *Gossypium hirsutum* | 32.39 | 121.05 | 2012 | 0 | 0 | 0 | 0 | 0 | 0 | 0 | 0 | 64 | 0 | 0 | 0 |
| 196 | Sanmenxia | Site1 | *Prunus davidiana* | 34.78 | 111.1 | 2012 | 0 | 0 | 0 | 0 | 0 | 0 | 0 | 0 | 0 | 0 | 0 | 3 |
| 197 |  | Site2 | *Prunusxia2idia* | 34.75 | 111.14 | 2011 | 0 | 0 | 0 | 0 | 0 | 0 | 0 | 0 | 0 | 0 | 0 | 33 |
| 198 | Shantou | Site1 | *Benincasa Savi* | 23.63 | 117.32 | 2013 | 0 | 0 | 1 | 0 | 0 | 0 | 0 | 48 | 0 | 0 | 0 | 0 |
| 199 | Shaoguan | Site1 | *Citrus reticulata Blanco* | 24.81 | 113.59 | 2014 | 0 | 0 | 0 | 0 | 0 | 0 | 0 | 0 | 0 | 0 | 40 | 0 |
| 200 |  |  | *Phaseolus vulgaris* | 24.81 | 113.59 | 2014 | 63 | 0 | 0 | 0 | 0 | 0 | 0 | 0 | 0 | 0 | 0 | 0 |
| 201 | Shenyang | Site1 | *Solanum melongena* | 41.88 | 123.39 | 2011 | 0 | 0 | 40 | 0 | 0 | 0 | 0 | 0 | 0 | 0 | 0 | 0 |
| 202 |  | Site2 | *Phaseolus vulgaris* | 41.88 | 123.59 | 2014 | 0 | 0 | 19 | 0 | 0 | 0 | 0 | 0 | 0 | 0 | 0 | 0 |
| 203 |  |  | *Solanum lycopersicum* | 41.88 | 123.59 | 2014 | 0 | 0 | 79 | 0 | 0 | 0 | 0 | 0 | 0 | 0 | 0 | 0 |
| 204 |  |  | *Solanum melongena* | 41.88 | 123.59 | 2014 | 0 | 0 | 50 | 1 | 0 | 0 | 0 | 0 | 0 | 0 | 0 | 0 |
| 205 |  |  | *Vigna unguiculata* | 41.88 | 123.59 | 2014 | 0 | 0 | 51 | 3 | 7 | 0 | 0 | 0 | 0 | 0 | 0 | 0 |
| 206 |  | Site3 | *Malus Chaenomeles* | 41.85 | 123.44 | 2016 | 0 | 10 | 0 | 0 | 0 | 0 | 0 | 0 | 0 | 0 | 0 | 0 |
| 207 |  |  | *Prunus persica f. rubro-plena* | 41.85 | 123.44 | 2016 | 0 | 0 | 0 | 0 | 0 | 0 | 0 | 0 | 0 | 0 | 0 | 27 |
| 208 |  | Site4 | *Ipomoea batatas* | 41.83 | 123.56 | 2015 | 0 | 0 | 0 | 3 | 0 | 0 | 0 | 0 | 0 | 0 | 0 | 0 |
| 209 |  |  | *Solanum lycopersicum* | 41.83 | 123.56 | 2015 | 0 | 0 | 44 | 0 | 0 | 0 | 0 | 0 | 0 | 0 | 0 | 0 |
| 210 |  | Site5 | *Solanum melongena* | 41.79 | 123.63 | 2009 | 0 | 0 | 79 | 0 | 0 | 0 | 0 | 0 | 0 | 0 | 0 | 0 |
| 211 |  |  | *Vigna unguiculata* | 41.79 | 123.63 | 2009 | 0 | 0 | 0 | 13 | 0 | 0 | 0 | 0 | 0 | 0 | 0 | 0 |
| 212 |  | Site6 | *Cucumis sativus* | 41.79 | 123.64 | 2012 | 0 | 0 | 55 | 0 | 0 | 0 | 0 | 0 | 0 | 0 | 0 | 0 |
| 213 |  | Site7 | *Vigna unguiculata* | 41.77 | 123.67 | 2008 | 0 | 0 | 3 | 0 | 0 | 0 | 0 | 0 | 0 | 0 | 0 | 0 |
| 214 | Shihezi | Site1 | *Armeniaca mume* | 44.29 | 85.98 | 2016 | 0 | 0 | 9 | 0 | 0 | 0 | 0 | 0 | 0 | 0 | 0 | 0 |
| 215 |  |  | *Gossypium spp* | 44.29 | 85.98 | 2016 | 0 | 0 | 15 | 0 | 0 | 0 | 0 | 0 | 0 | 0 | 0 | 0 |
| 216 |  | Site2 | *Gossypium hirsutum* | 44.28 | 86 | 2013 | 0 | 3 | 0 | 0 | 0 | 0 | 0 | 0 | 0 | 0 | 0 | 0 |
| 217 | Shijiazhuang | Site1 | *Solanum melongena* | 38.13 | 114.45 | 2011 | 0 | 0 | 50 | 0 | 0 | 0 | 0 | 0 | 0 | 0 | 0 | 0 |
| 218 | Shilihe | Site1 | *Capsicum annuum* | 41.62 | 123.31 | 2014 | 0 | 0 | 2 | 1 | 0 | 0 | 0 | 0 | 0 | 0 | 0 | 0 |
| 219 |  |  | *Cucumis sativus* | 41.62 | 123.31 | 2014 | 0 | 0 | 47 | 0 | 0 | 0 | 0 | 0 | 0 | 0 | 0 | 0 |
| 220 |  |  | *Solanum lycopersicum* | 41.62 | 123.31 | 2014 | 0 | 0 | 27 | 0 | 0 | 0 | 0 | 0 | 0 | 0 | 0 | 0 |
| 221 |  |  | *Solanum melongena* | 41.62 | 123.31 | 2014 | 0 | 0 | 25 | 0 | 0 | 0 | 0 | 0 | 0 | 0 | 0 | 0 |
| 222 |  |  | *Vigna unguiculata* | 41.62 | 123.31 | 2014 | 0 | 0 | 3 | 0 | 0 | 0 | 0 | 0 | 0 | 0 | 0 | 0 |
| 223 | Shimen | Site1 | *Ipomoea aquatica* | 29.61 | 111.4 | 2014 | 0 | 0 | 50 | 0 | 0 | 0 | 0 | 0 | 0 | 0 | 0 | 0 |
| 224 | Shishi | Site1 | *Arachis hypogaea* | 24.73 | 118.71 | 2013 | 0 | 0 | 67 | 0 | 0 | 0 | 0 | 0 | 0 | 0 | 0 | 0 |
| 225 | Shouguang | Site1 | *Gossypium hirsutum* | 36.87 | 118.72 | 2011 | 0 | 0 | 91 | 0 | 0 | 0 | 0 | 0 | 0 | 0 | 0 | 0 |
| 226 |  | Site2 | *Gossypium hirsutum* | 36.85 | 118.72 | 2012 | 0 | 0 | 3 | 0 | 0 | 0 | 0 | 0 | 0 | 0 | 0 | 0 |
| 227 | Suzhou | Site1 | *Solanum melongena* | 31.39 | 120.55 | 2010 | 0 | 0 | 1 | 0 | 0 | 0 | 0 | 0 | 0 | 0 | 0 | 0 |
| 228 | Taian | Site1 | *Malus domestica* | 36.24 | 117.11 | 2012 | 0 | 35 | 0 | 0 | 0 | 0 | 0 | 0 | 0 | 0 | 0 | 30 |
| 229 |  | Site2 | *Glycine max* | 36.18 | 117.2 | 2014 | 0 | 0 | 3 | 0 | 0 | 0 | 0 | 0 | 0 | 0 | 0 | 0 |
| 230 |  |  | *Solanum lycopersicum* | 36.18 | 117.2 | 2014 | 62 | 0 | 1 | 0 | 0 | 0 | 0 | 0 | 0 | 0 | 0 | 0 |
| 231 | Taigu | Site1 | *Solanum lycopersicum* | 37.4 | 112.5 | 2014 | 0 | 0 | 3 | 0 | 0 | 0 | 0 | 0 | 0 | 0 | 0 | 0 |
| 232 |  |  | *Solanum melongena* | 37.4 | 112.5 | 2014 | 0 | 0 | 60 | 0 | 0 | 0 | 0 | 0 | 0 | 0 | 0 | 0 |
| 233 | Taihu | Site1 | *Gossypium hirsutum* | 30.39 | 112.26 | 2012 | 0 | 0 | 0 | 0 | 0 | 0 | 0 | 48 | 0 | 0 | 0 | 0 |
| 234 | Tianshui | Site1 | *Glycine max* | 34.57 | 105.64 | 2008 | 22 | 0 | 0 | 0 | 0 | 0 | 0 | 0 | 0 | 0 | 0 | 0 |
| 235 |  |  | *Malus domestica* | 34.57 | 105.64 | 2008 | 0 | 43 | 0 | 0 | 0 | 0 | 0 | 0 | 0 | 0 | 0 | 0 |
| 236 | Tongan | Site1 | *Phaseolus vulgaris* | 24.75 | 118.13 | 2013 | 0 | 0 | 48 | 0 | 0 | 0 | 0 | 0 | 0 | 0 | 0 | 0 |
| 237 | Weifang | Site1 | *Glycine max* | 36.74 | 119.21 | 2015 | 0 | 0 | 3 | 0 | 0 | 0 | 0 | 0 | 0 | 0 | 0 | 0 |
| 238 |  |  | *Ipomoea batatas* | 36.74 | 119.21 | 2015 | 0 | 0 | 3 | 0 | 0 | 0 | 0 | 0 | 0 | 0 | 0 | 0 |
| 239 |  |  | *Solanum lycopersicum* | 36.74 | 119.21 | 2015 | 0 | 0 | 13 | 0 | 0 | 0 | 0 | 0 | 0 | 0 | 0 | 0 |
| 240 | Weiweicun | Site1 | *Cucurbita moschata* | 29.19 | 121.7 | 2016 | 0 | 0 | 0 | 0 | 0 | 0 | 0 | 0 | 30 | 0 | 0 | 0 |
| 241 | Weixi | Site1 | *Citrullus lanatus* | 27.4 | 99.07 | 2013 | 0 | 0 | 0 | 3 | 0 | 0 | 0 | 0 | 0 | 0 | 0 | 0 |
| 242 |  |  | *Phaseolus vulgaris* | 27.4 | 99.07 | 2013 | 0 | 0 | 0 | 26 | 0 | 0 | 0 | 0 | 0 | 0 | 0 | 0 |
| 243 |  |  | *Pseudocydonia sinensiso* | 27.4 | 99.07 | 2013 | 0 | 0 | 0 | 5 | 0 | 0 | 0 | 0 | 0 | 0 | 0 | 0 |
| 244 | Wenzhou | Site1 | *Phaseolus vulgaris* | 28.01 | 120.72 | 2015 | 0 | 0 | 0 | 0 | 0 | 0 | 0 | 0 | 3 | 0 | 0 | 0 |
| 245 | Wuhan | Site1 | *Gossypium spp* | 30.74 | 114.42 | 2008 | 0 | 0 | 3 | 0 | 0 | 0 | 0 | 0 | 0 | 0 | 0 | 0 |
| 246 | Wuxi | Site1 | *Citrus reticulata Blanco* | 31.92 | 120.28 | 2015 | 0 | 0 | 0 | 0 | 0 | 0 | 0 | 0 | 0 | 0 | 64 | 0 |
| 247 | Wuyishan | Site1 | *Amygdalus persica* | 27.74 | 118.04 | 2017 | 0 | 0 | 3 | 0 | 0 | 0 | 0 | 0 | 0 | 0 | 0 | 0 |
| 248 |  |  | *Colocasia esculenta* | 27.74 | 118.04 | 2017 | 0 | 0 | 0 | 0 | 0 | 0 | 0 | 0 | 16 | 0 | 0 | 0 |
| 249 |  |  | *Glycine max* | 27.74 | 118.04 | 2017 | 0 | 0 | 27 | 0 | 0 | 0 | 0 | 0 | 0 | 0 | 0 | 0 |
| 250 |  |  | *Ipomoea batatas* | 27.74 | 118.04 | 2017 | 0 | 0 | 0 | 0 | 0 | 0 | 0 | 0 | 24 | 0 | 0 | 0 |
| 251 |  |  | *Sesamum indicum* | 27.74 | 118.04 | 2017 | 0 | 0 | 0 | 0 | 11 | 0 | 0 | 0 | 0 | 0 | 0 | 0 |
| 252 |  |  | *Thalia dealbata* | 27.74 | 118.04 | 2017 | 0 | 0 | 0 | 0 | 0 | 0 | 0 | 0 | 39 | 0 | 0 | 0 |
| 253 | Xi'an | Site1 | *Malus domestica* | 34.32 | 108.89 | 2008 | 0 | 0 | 3 | 0 | 0 | 0 | 0 | 0 | 0 | 0 | 0 | 0 |
| 254 | Xiayi | Site1 | *Gossypium hirsutum* | 34.24 | 116.1 | 2012 | 0 | 0 | 42 | 0 | 0 | 0 | 0 | 0 | 0 | 0 | 0 | 0 |
| 255 | Xingcheng | Site1 | *Malus domestica* | 40.65 | 120.74 | 2008 | 0 | 36 | 0 | 0 | 0 | 0 | 0 | 0 | 0 | 0 | 0 | 0 |
| 256 |  | Site2 | *Malus domestica* | 40.32 | 120.7 | 2008 | 0 | 3 | 36 | 0 | 0 | 0 | 0 | 0 | 0 | 0 | 0 | 0 |
| 257 | Xingtai | Site1 | *Solanum melongena* | 37.01 | 114.61 | 2010 | 0 | 0 | 28 | 0 | 0 | 0 | 0 | 0 | 0 | 0 | 0 | 0 |
| 258 | Xining | Site1 | *Livistona chinensis* | 36.58 | 101.73 | 2010 | 3 | 0 | 54 | 0 | 0 | 0 | 0 | 0 | 0 | 0 | 0 | 0 |
| 259 |  | Site2 | *Alcea rosea* | 36.56 | 101.77 | 2011 | 0 | 0 | 0 | 31 | 0 | 0 | 0 | 0 | 0 | 0 | 0 | 0 |
| 260 | Xuzhou | Site1 | *Gossypium hirsutum* | 34.24 | 117.33 | 2011 | 0 | 0 | 22 | 0 | 0 | 0 | 0 | 0 | 0 | 0 | 0 | 0 |
| 261 |  | Site2 | *Pyrus spp* | 34.17 | 117.24 | 2009 | 0 | 0 | 0 | 0 | 0 | 0 | 0 | 0 | 3 | 0 | 0 | 0 |
| 262 |  |  | *Trifolium repens* | 34.17 | 117.24 | 2009 | 0 | 0 | 0 | 3 | 0 | 0 | 0 | 0 | 0 | 0 | 0 | 0 |
| 263 |  | Site3 | *Gossypium hirsutum* | 34.15 | 117.24 | 2012 | 0 | 0 | 67 | 0 | 0 | 0 | 0 | 0 | 0 | 0 | 0 | 0 |
| 264 |  | Site4 | *Phaseolus vulgaris* | 34.1 | 117.19 | 2015 | 0 | 0 | 10 | 1 | 0 | 0 | 0 | 0 | 0 | 0 | 0 | 0 |
| 265 |  |  | *Solanum melongena* | 34.1 | 117.19 | 2015 | 0 | 0 | 3 | 0 | 0 | 0 | 0 | 0 | 0 | 0 | 0 | 0 |
| 266 | Yaan | Site1 | *Citrus reticulata Blanco* | 29.35 | 102.64 | 2016 | 0 | 0 | 0 | 0 | 0 | 0 | 0 | 0 | 0 | 0 | 40 | 0 |
| 267 |  |  | *Lycopersicon esculentum* | 29.35 | 102.64 | 2016 | 0 | 0 | 0 | 0 | 0 | 0 | 0 | 0 | 0 | 80 | 0 | 0 |
| 268 |  |  | *Solanum melongena* | 29.35 | 102.64 | 2016 | 0 | 0 | 0 | 0 | 0 | 0 | 0 | 0 | 0 | 8 | 0 | 0 |
| 269 | Yancheng | Site1 | *Prunus ceraifera* | 33.38 | 120.08 | 2011 | 0 | 0 | 0 | 0 | 0 | 0 | 0 | 0 | 0 | 0 | 0 | 33 |
| 270 | Yandong | Site1 | *Gossypium hirsutum* | 33.36 | 120.27 | 2012 | 0 | 0 | 0 | 0 | 0 | 0 | 0 | 0 | 53 | 0 | 0 | 0 |
| 271 | Yangling | Site1 | *Amygdalus persicas* | 34.27 | 108.07 | 2016 | 0 | 0 | 0 | 0 | 0 | 0 | 0 | 0 | 0 | 0 | 0 | 6 |
| 272 |  |  | *Malus baccata* | 34.27 | 108.07 | 2016 | 0 | 0 | 0 | 0 | 0 | 0 | 0 | 0 | 0 | 0 | 0 | 6 |
| 273 |  |  | *Malus pumila* | 34.27 | 108.07 | 2016 | 0 | 0 | 0 | 0 | 0 | 0 | 0 | 0 | 0 | 0 | 0 | 10 |
| 274 |  |  | *Pharbitis nil* | 34.27 | 108.07 | 2016 | 0 | 0 | 0 | 0 | 0 | 0 | 0 | 0 | 0 | 0 | 0 | 3 |
| 275 |  |  | *Phaseolus vulgaris* | 34.27 | 108.07 | 2016 | 0 | 0 | 0 | 0 | 0 | 0 | 0 | 0 | 0 | 0 | 0 | 3 |
| 276 |  |  | *Vaccinium Spp* | 34.27 | 108.07 | 2016 | 0 | 0 | 0 | 0 | 0 | 0 | 0 | 0 | 0 | 0 | 0 | 11 |
| 277 | Yangzhou | Site1 | *Glycine max* | 32.45 | 119.44 | 2011 | 0 | 0 | 3 | 0 | 0 | 0 | 0 | 0 | 0 | 0 | 0 | 0 |
| 278 |  | Site2 | *Glycine max* | 32.33 | 119.41 | 2010 | 0 | 0 | 3 | 0 | 0 | 0 | 0 | 0 | 0 | 0 | 0 | 0 |
| 279 | Yanji | Site1 | *Glycine max* | 45.43 | 128.3 | 2014 | 0 | 0 | 0 | 7 | 0 | 0 | 0 | 0 | 0 | 0 | 0 | 0 |
| 280 |  | Site2 | *Glycine max* | 42.88 | 129.47 | 2011 | 0 | 0 | 3 | 0 | 0 | 0 | 0 | 0 | 0 | 0 | 0 | 0 |
| 281 |  |  | *Oxalis comicalata* | 42.88 | 129.47 | 2011 | 7 | 0 | 29 | 1 | 0 | 0 | 0 | 0 | 0 | 0 | 0 | 0 |
| 282 |  |  | *Phaseolus vulgaris* | 42.88 | 129.47 | 2011 | 0 | 0 | 40 | 0 | 0 | 0 | 0 | 0 | 0 | 0 | 0 | 0 |
| 283 | Yanliang | Site1 | *Malus domestica* | 34.66 | 109.27 | 2008 | 0 | 0 | 6 | 0 | 0 | 0 | 0 | 0 | 0 | 0 | 0 | 0 |
| 284 | Yanshou | Site1 | *Glycine max* | 45.46 | 128.39 | 2017 | 0 | 0 | 27 | 0 | 0 | 0 | 0 | 0 | 0 | 0 | 0 | 0 |
| 285 |  |  | *Phaseolus vulgaris* | 45.46 | 128.39 | 2017 | 0 | 0 | 3 | 0 | 0 | 0 | 0 | 0 | 0 | 0 | 0 | 0 |
| 286 |  |  | *Solanum melongena* | 45.46 | 128.39 | 2017 | 0 | 0 | 15 | 0 | 0 | 0 | 0 | 0 | 0 | 0 | 0 | 0 |
| 287 |  |  | *Vigna radiata* | 45.46 | 128.39 | 2017 | 0 | 0 | 0 | 28 | 0 | 0 | 0 | 0 | 0 | 0 | 0 | 0 |
| 288 |  |  | *Zea mays* | 45.46 | 128.39 | 2017 | 0 | 0 | 18 | 0 | 0 | 0 | 0 | 0 | 0 | 0 | 0 | 0 |
| 289 |  | Site2 | *Glycine max* | 45.44 | 128.37 | 2014 | 0 | 0 | 0 | 3 | 0 | 0 | 0 | 0 | 0 | 0 | 0 | 0 |
| 290 | Yichun | Site1 | *Vigna unguiculata* | 47.73 | 128.78 | 2008 | 0 | 0 | 3 | 0 | 0 | 0 | 0 | 0 | 0 | 0 | 0 | 0 |
| 291 | Yining | Site1 | *Gossypium herbaceum* | 43.98 | 81.49 | 2008 | 0 | 3 | 0 | 0 | 0 | 0 | 0 | 0 | 0 | 0 | 0 | 0 |
| 292 |  |  | *Malus domestica* | 43.98 | 81.49 | 2008 | 0 | 3 | 0 | 0 | 0 | 0 | 0 | 0 | 0 | 0 | 0 | 0 |
| 293 | Yongfu | Site1 | *Benincasa Savi* | 24.98 | 109.99 | 2013 | 0 | 0 | 0 | 27 | 0 | 0 | 0 | 0 | 0 | 0 | 0 | 0 |
| 294 |  |  | *Colocasia esculenta* | 24.98 | 109.99 | 2013 | 0 | 0 | 0 | 43 | 0 | 0 | 0 | 0 | 0 | 0 | 0 | 0 |
| 295 | Yongjing | Site1 | *Fragaria sculentalancop* | 35.95 | 103.27 | 2016 | 0 | 3 | 0 | 0 | 0 | 0 | 0 | 0 | 0 | 0 | 0 | 0 |
| 296 |  | Site2 | *Glycine max* | 35.93 | 103.27 | 2016 | 0 | 0 | 3 | 0 | 0 | 0 | 0 | 0 | 0 | 0 | 0 | 0 |
| 297 | Yulong | Site1 | *Glycine max* | 26.93 | 100.22 | 2008 | 0 | 0 | 0 | 14 | 0 | 0 | 0 | 0 | 0 | 0 | 0 | 0 |
| 298 |  | Site2 | *Glycine max* | 26.89 | 100.22 | 2009 | 0 | 0 | 0 | 0 | 0 | 0 | 0 | 0 | 32 | 0 | 0 | 0 |
| 299 | Yuncheng | Site1 | *Glycine max* | 35.06 | 110.99 | 2010 | 0 | 0 | 0 | 0 | 0 | 40 | 0 | 0 | 0 | 0 | 0 | 0 |
| 300 |  |  | *Phaseolus vulgaris* | 35.06 | 110.99 | 2010 | 0 | 0 | 0 | 0 | 0 | 3 | 0 | 0 | 0 | 0 | 0 | 0 |
| 301 |  | Site2 | *Gossypium spp* | 35.01 | 110.96 | 2008 | 0 | 0 | 3 | 0 | 0 | 0 | 0 | 0 | 0 | 0 | 0 | 0 |
| 302 | Yuxi | Site1 | *Phaseolus vulgaris* | 24.34 | 102.51 | 2014 | 0 | 0 | 0 | 28 | 0 | 0 | 0 | 0 | 0 | 0 | 0 | 0 |
| 303 | Zhangjiajie | Site1 | *Cucumis sativus* | 29.26 | 110.47 | 2013 | 0 | 0 | 11 | 0 | 0 | 0 | 0 | 0 | 0 | 0 | 0 | 0 |
| 304 |  |  | *Phaseolus vulgaris* | 29.26 | 110.47 | 2013 | 0 | 0 | 3 | 38 | 0 | 0 | 0 | 0 | 0 | 0 | 0 | 0 |
| 305 | Zhangye | Site1 | *Zea mays* | 38.99 | 100.44 | 2016 | 0 | 0 | 8 | 0 | 0 | 0 | 0 | 0 | 0 | 0 | 0 | 0 |
| 306 | Zhangzhou | Site1 | *Ficus carica* | 24.49 | 117.64 | 2016 | 0 | 0 | 9 | 0 | 0 | 0 | 0 | 0 | 0 | 0 | 0 | 0 |
| 307 |  |  | *Glycine max* | 24.49 | 117.64 | 2016 | 0 | 0 | 0 | 3 | 0 | 0 | 0 | 0 | 0 | 0 | 0 | 0 |
| 308 |  |  | *Musa basjoo Siebold* | 24.49 | 117.64 | 2016 | 0 | 0 | 0 | 0 | 18 | 0 | 0 | 0 | 0 | 0 | 0 | 0 |
| 309 |  |  | *Pharbitis nil* | 24.49 | 117.64 | 2016 | 0 | 0 | 3 | 0 | 0 | 0 | 0 | 0 | 0 | 0 | 0 | 0 |
| 310 | Zhanjiang | Site1 | *Phaseolus vulgaris* | 21.12 | 110.26 | 2015 | 4 | 0 | 0 | 0 | 0 | 0 | 0 | 0 | 8 | 0 | 0 | 0 |
| 311 | Zhengzhou | Site1 | *Gossypium hirsutum* | 34.79 | 113.66 | 2012 | 0 | 0 | 42 | 0 | 0 | 0 | 0 | 0 | 2 | 0 | 0 | 0 |
| 312 |  | Site2 | *Gossypium spp* | 34.6 | 113.6 | 2008 | 0 | 0 | 5 | 0 | 0 | 0 | 0 | 0 | 0 | 0 | 0 | 0 |
| 313 |  |  | *Malus domestica* | 34.6 | 113.6 | 2008 | 44 | 0 | 0 | 0 | 0 | 0 | 0 | 0 | 0 | 0 | 0 | 0 |
| 314 | Zhenjiang | Site1 | *Glycine max* | 32.11 | 119.45 | 2008 | 0 | 0 | 34 | 0 | 0 | 0 | 0 | 0 | 0 | 0 | 0 | 0 |
| 315 |  | Site1 | *Amaranthus mangostanus* | 32.11 | 119.43 | 2015 | 0 | 0 | 4 | 0 | 0 | 0 | 0 | 0 | 0 | 0 | 0 | 0 |
| 316 |  |  | *Solanum melongena* | 32.11 | 119.43 | 2015 | 0 | 0 | 9 | 1 | 0 | 0 | 0 | 0 | 0 | 0 | 0 | 0 |
| 317 |  |  | *Vigna unguiculata* | 32.11 | 119.43 | 2015 | 0 | 0 | 2 | 9 | 0 | 0 | 0 | 0 | 0 | 0 | 0 | 0 |
| 318 |  |  | *Zea mays* | 32.11 | 119.43 | 2015 | 0 | 0 | 20 | 0 | 0 | 0 | 0 | 0 | 0 | 0 | 0 | 0 |

Abbreviations: N, sample size; Tur, *Tetranychus urticae* (red form); Tug, *T. urticae* (green form); Ttr, *T. truncatus*; Tpu, *T. pueraricola*; Tpi, *T. piercei*; Tph, *T. phaselus*; Tma, *T. macfarlanei*; Tlu, *T. ludei*; Tka, *T. kanzawai*; Tev, *T. evansi*; Pci, *Panonychus citri*; Avi, *Amphitetranychus viennensis*.

**Table S2** GenBank accession of sequences used in phylogenetic reconstruction at the global scale^*^

| Genus | Speccies | CO1 | 18S | 28S |
| --- | --- | --- | --- | --- |
| *Bryobia* | *B. eharai* Pritchard & Keifer | - | AB926227 | AB926318 |
|  | *B. praetiosa* Koch | AB981203 | AB926228 | AB926319 |
|  | *P. latens* (Muller) | AB981204 | AB926229 | AB926320 |
| *Tetranychina* | *T. harti* (Ewing) | GQ141920 ^(^**^1)^** | AB926230 | AB926321 |
| *Eurytetranychoides* | *E. japonicus* (Ehara) | AB981205 | AB926231 | AB926322 |
| *Eutetranychus* | *E. africanus* (Tucker) | - | AB926232 | AB926323 |
| *Aponychus* | *A. corpuzae* Rimando | AB981206 | AB926233 | AB926324 |
|  | *A. firmianae* (Ma & Yuan) | - | AB926234 | AB926325 |
| *Panonychus* | *P. bambusicola* Ehara & Gotoh | AB981207 | AB926235 | AB926326 |
|  | *P. caglei* Mellot | KC502920**^(2)^** | AB926236 | AB926327 |
|  | *P. citri* (McGregor) | AB981208 | AB926237 | AB926328 |
|  | *P. elongatus* Manson | - | AB926238 | AB926329 |
|  | *P. mori* Yokoyama | AB981209 | AB926239 | AB926330 |
|  | *P. osmanthi* Ehara & Gotoh | AB981210 | AB926240 | AB926331 |
|  | *P. thelytokus* Ehara & Gotoh | AB981211 | AB926241 | AB926332 |
|  | *P. ulmi* (Koch) | AB981212 | AB926242 | AB926333 |
| *Sasanychus* | *S. akitanus* (Ehara) | AB981213 | AB926243 | AB926334 |
|  | *S. pusillus* Ehara & Gotoh | AB981214 | AB926244 | AB926335 |
| *Schizotetranychus* | *S. bambusae* Reck | AB981215 | AB926245 | AB926336 |
|  | *S. brevisetosus* Ehara | AB981216 | AB926246 | AB926337 |
|  | *S. cercidiphyll*i Ehara | AB981217 | AB926247 | AB926338 |
|  | *S. gilvus* Ehara & Ohashi | AB981218 | AB926248 | AB926339 |
|  | *S. lespedezae* Begljarov & Mitrofanov | AB981219 | AB926249 | AB926340 |
|  | *S. rec*ki Ehara | AB981220 | AB926250 | AB926341 |
|  | *S. schizopus* (Zacher) | AB981221 | AB926251 | AB926342 |
|  | *S. shii* (Ehara) | AB981222 | AB926252 | AB926343 |
| *Stigmaeopsis* | *S. celarius* Banks | AB981223 | AB926253 | AB926344 |
|  | *S. longus* (Saito) | AB981224 | AB926254 | AB926345 |
|  | *S. miscanth*i (Saito) | AB981225 | AB926255 | AB926346 |
|  | *S. saharai* Saito & Mori | AB981226 | AB926256 | AB926347 |
|  | *S. takahashii* Saito & Mori | AB981227 | AB926257 | AB926348 |
| *Yezonychus* | *Y. sapporensis* Ehara | AB981228 | AB926258 | AB926349 |
| *Eotetranychus* | *E. asiaticus* Ehara | AB981229 | AB926259 | AB926350 |
|  | *E. boreus* Ehara | - | AB926260 | AB926351 |
|  | *E. celtis* Ehara | AB981230 | AB926261 | AB926352 |
|  | *E. cornicola* Ehara | AB981231 | AB926262 | AB926353 |
|  | *E. dissectus* Ehara | AB981232 | AB926263 | AB926354 |
|  | *E. nomurai* Ehara | AB981233 | AB926264 | AB926355 |
|  | *E. pruni* (Oudemans) | - | AB926265 | AB926356 |
|  | *E. querci* Reeves | - | AB926266 | AB926357 |
|  | *E. quercifoliae* Ehara & Gotoh | AB981234 | AB926267 | AB926358 |
|  | *E. rubricans* Ehara | - | AB926268 | AB926359 |
|  | *E. smithi* Pritchard & Baker | AB981235 | AB926269 | AB926360 |
|  | *E. spectabilis* Ehara | - | AB926270 | AB926361 |
|  | E*. suginamensis*(Yokoyama) | AB981236 | AB926271 | AB926362 |
|  | *E. tiliarium* (Hermann) | X80864**^(3)^** | AB926272 | AB926363 |
|  | *E. toyoshimai* Ehara & Gotoh | - | AB926273 | AB926364 |
|  | *E. uchida*i Ehara | AB981237 | AB926274 | AB926365 |
|  | *E. uncatus* Garman | - | AB926275 | AB926366 |
| *Oligonychus* | *O. amiensis* Ehara & Gotoh | AB683672 | AB926276 | AB926367 |
|  | *O. biharensis* (Hirst) | AB683678 | AB926277 | AB926368 |
|  | *O. camelliae* Ehara & Gotoh | AB683662 | AB926278 | AB926369 |
|  | *O. castaneae* Ehara & Gotoh | AB683667 | AB926279 | AB926370 |
|  | *O. clavatus* (Ehara) | AB683654 | AB926280 | AB926371 |
|  | *O. coffeae* (Nietner) | AB683670 | AB926281 | AB926372 |
|  | *O. gotohi* Ehara | AB683668 | AB926282 | AB926373 |
|  | *O. hondoensis* (Ehara) | AB683658 | AB926283 | AB926374 |
|  | *O. ilicis* (McGregor) | AB683660 | AB926284 | AB926375 |
|  | *O. karamatus* (Ehara) | AB683656 | AB926285 | AB926376 |
|  | *O. modestus* (Banks) | AB683677 | AB926286 | AB926377 |
|  | *O. orthius* Rimando | AB683675 | AB926287 | AB926378 |
|  | *O. perditus* Pritchard & Baker | AB683665 | AB926288 | AB926379 |
|  | *O. pustulosus* Ehara | AB683655 | AB926289 | AB926380 |
|  | *O. rubicundus* Ehara | AB683681 | AB926290 | AB926381 |
|  | *O. ununguis* (Jacobi) | AB683664 | AB926291 | AB926382 |
| Amphitetranychus | *A. quercivorus* (Ehara & Gotoh) | AB981238 | AB926292 | AB926383 |
|  | *A. viennensis* (Zacher) | AB981239 | AB926293 | AB926384 |
| Tetranychus | *T. bambusae* Wang & Ma | KR269716**^(4)^** | AB926294 | AB926385 |
|  | *T. evansi* Baker & Pritchard | AB736039 | AB926295 | AB926386 |
|  | *T. ezoensis* Ehara | AB736042 | AB926296 | AB926387 |
|  | *T. huhhotensis* Ehara, Gotoh & Hong | - | AB926297 | AB926388 |
|  | *T. kanzawai* Kishida | AB736043 | AB926298 | AB926389 |
|  | *T. lombardinii* Baker & Pritchard | | AB926299 | AB926390 |
|  | *T. ludeni* Zacher | AB736051 | AB926300 | AB926391 |
|  | *T. macfarlane*i Baker & Pritchard | **KJ729019^(5)^** | AB926301 | AB926392 |
|  | *T. mergans*er Boudreau | KM596707**^(6)^** | AB926302 | AB926393 |
|  | *T. misumaiensis* Ehara & Gotoh | AB736054 | AB926303 | AB926394 |
|  | *T. neocaledonicus* Andr | AB736055 | AB926304 | AB926395 |
|  | *T. okinawanus* Ehara | AB736058 | AB926305 | AB926396 |
|  | *T. parakanzawai* Ehara | AB736060 | AB926306 | AB926397 |
|  | *T. phaselus* Ehara | AB736066 | AB926307 | AB926398 |
|  | *T. piercei* McGregor | AB736068 | AB926308 | AB926399 |
|  | *T. pueraricola* Ehara & Gotoh | AB736071 | AB926309 | AB926400 |
|  | *T. truncatus* Ehara | AB736075 | AB926310 | AB926401 |
|  | *T. turkestani* Ugarov & Nikolski | AB981240 | AB926311 | AB926402 |
|  | *T. urticae* Koch (green form) | AB736076 | AB926312 | AB926403 |
|  | *T. urticae* Koch (red form) | AB736079 | AB926313 | AB926404 |
|  | *T. zeae* Ehara, Gotoh & Hong | - | AB926314 | AB926405 |

*Most sequences used in this study were from Matsuda et al. (2014). Others who have contributed samples and sequences include: ^(1)^ Hong X-Y and Li G-Q 2009; ^(2)^ Khaing TM and Lee K-Y 2013; ^(3)^ Navajas M et al. 1996; ^(4)^ Srinivas N et al. 2015; ^(5)^ Chen D-S et al. 2014; ^(6)^ Otero-Colina G, and Segura-Leon OL, 2014.

**REFERENCES**

Chen D-S, Jin P-Y, Zhang K-J, Ding X-L, Yang S-X, Ju, J-F, Zhao J-Y, Hong X-Y (2014) The complete mitochondrial genomes of six species of *Tetranychus* provide insights into the phylogeny and evolution of spider mites. *PLoS ONE*, **9**, e110625.

Matsuda T, Morishita M, Hinomoto N, Gotoh T (2014) Phylogenetic analysis of the spider mite sub-family Tetranychinae (Acari: Tetranychidae) based on the mitochondrial COI gene and the 18S and the 59 end of the 28S rRNA genes indicates that several genera are polyphyletic. *PLoS ONE*, **9**, e108672.

Navajas M, Gutierrez J, Lagnel J, Boursot P (1996) Mitochondrial cytochrome oxidase I in tetranychid mites: a comparison between molecular phylogeny and changes of morphological and life history traits. *Bulletin of Entomological Research*, **86**, 407-417.

**Table S3** Relative abundance, host range and distribution of each spider mite species in China

|  | Relative abundance | | Host range | | Distribution |
| --- | --- | --- | --- | --- | --- |
| Species | NOC | AF value ± SE | HS | HF | latitudinal span (degrees latitude) |
| Tur | 413 | 0.0621 ± 0.0164 | 13 | 8 | 24.22 |
| Tug | 284 | 0.0546 ± 0.0159 | 8 | 5 | 22.96 |
| Ttr | 3734 | 0.4528 ± 0.0345 | 33 | 13 | 29.49 |
| Tpu | 1140 | 0.1402 ± 0.0234 | 27 | 10 | 22.55 |
| Tpi | 118 | 0.0241 ± 0.0097 | 8 | 5 | 12.92 |
| Tph | 88 | 0.0114 ± 0.0078 | 3 | 2 | 10.78 |
| Tma | 45 | 0.0056 ± 0.0056 | 1 | 1 | 0.00 |
| Tlu | 125 | 0.0186 ± 0.0096 | 5 | 5 | 1.87 |
| Tka | 751 | 0.1184 ± 0.0225 | 20 | 9 | 24.55 |
| Tev | 132 | 0.0094 ± 0.0052 | 3 | 2 | 6.26 |
| Pci | 369 | 0.0335 ± 0.0127 | 1 | 1 | 9.10 |
| Avi | 397 | 0.0692 ± 0.0175 | 14 | 5 | 17.62 |

Abbreviations: Tur, *Tetranychus urticae* (red form); Tug, *T. urticae* (green form); Ttr, *T. truncatus*; Tpu, *T. pueraricola*; Tpi, *T. piercei*; Tph, *T. phaselus*; Tma, *T. macfarlanei*; Tlu, *T. ludeni*; Tka, *T. kanzawai*; Tev, *T. evansi*; Pci, *Panonychus citri*; Avi, *Amphitetranychus viennensis.* NOC, Number of occurred times for each species. AF value, average frequency of occurrence for each species. HS, host species record number. HF, host family records number.

**Table S4** Mantel tests of association between genetic distance and species abundance, distribution range and host range

|  |  | Mantel test | |
| --- | --- | --- | --- |
|  |  | r | p value |
| China | Relative abundance | 0.282 | 0.119 |
|  | Host range | 0.675 | 0.003 |
|  | Latitudinal span | 0.661 | 0.001 |
| Global | Records number | 0.230 | 0.001 |
|  | Host range | 0.222 | 0.001 |
|  | Number of distributed country | 0.107 | 0.004 |

**Table S5** Pearson correlations between the species occurrence patterns and genetic distance to the focal species of different genera

|  | Occurrence number of historical records | | | Number of countries | | | | |
| --- | --- | --- | --- | --- | --- | --- | --- | --- |
| Genus | N | r | p |  | N | r | p |  |
| *Tetranychus* | 19 | - 0.263 | 0.275 |  | 19 | - 0.235 | 0.333 |  |
| *Eotetranychu* | 18 | - 0.469 | 0.049 |  | 18 | - 0.385 | 0.113 |  |
| *Panonychus* | 13 | - 0.673 | 0.017 |  | 13 | - 0.584 | 0.021 |  |
| *Oligonychus* | 12 | - 0.675 | 0.016 |  | 12 | - 0.675 | 0.046 |  |

Note: N, species number used in the analysis.

**Fig. S1** Bayesian tree with posterior probabilities (a) and RAxML ML tree with bootstrap percentages from 1000 rapid bootstrap replicates (b) for *Tetranychus* species in China**.** Numbers at branches represent Bayesian posterior probabilities (a) and maximum likelihood bootstrap percentages (b)


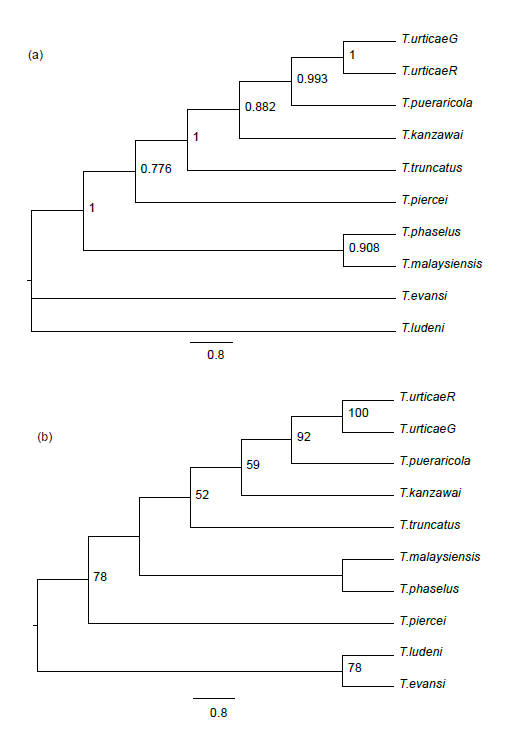


**Fig. S2** Bayesian tree with posterior probabilities of 88 species. Phylogenetic tree inferred from three combined DNA fragments (COI, 18S and 28s) using Bayesian method in MrBayes 3.2.6. Numbers at branches represent posterior probability values above 0.5


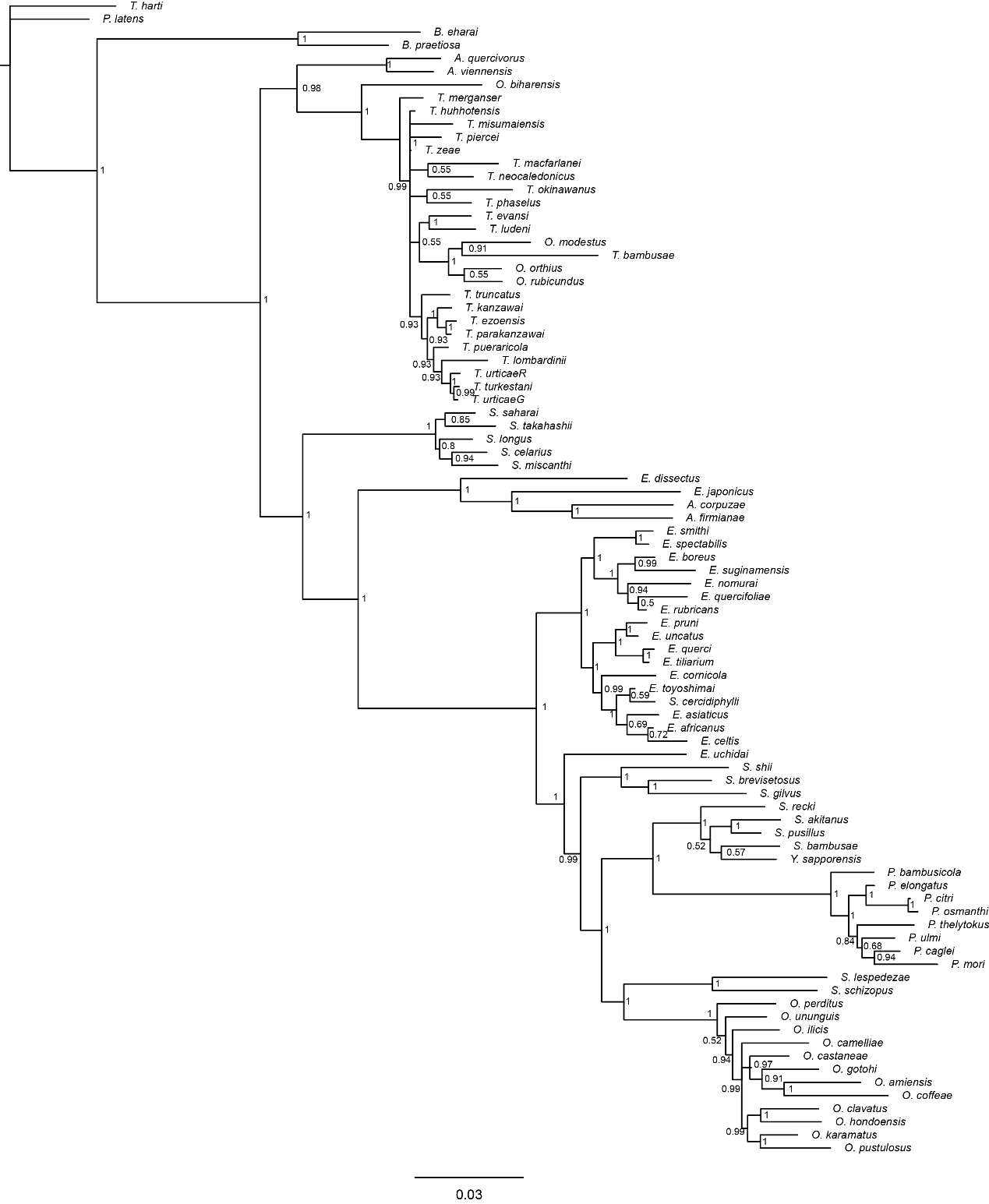


**Fig. S3** ML tree based on 1000 rapid bootstrap replicates of 88 species. Phylogenetic tree inferred from three combined DNA fragments (COI, 18S and 28s) in RaxmlGUI1.3. Numbers at branches represent maximum likelihood bootstrap percentages above 50%


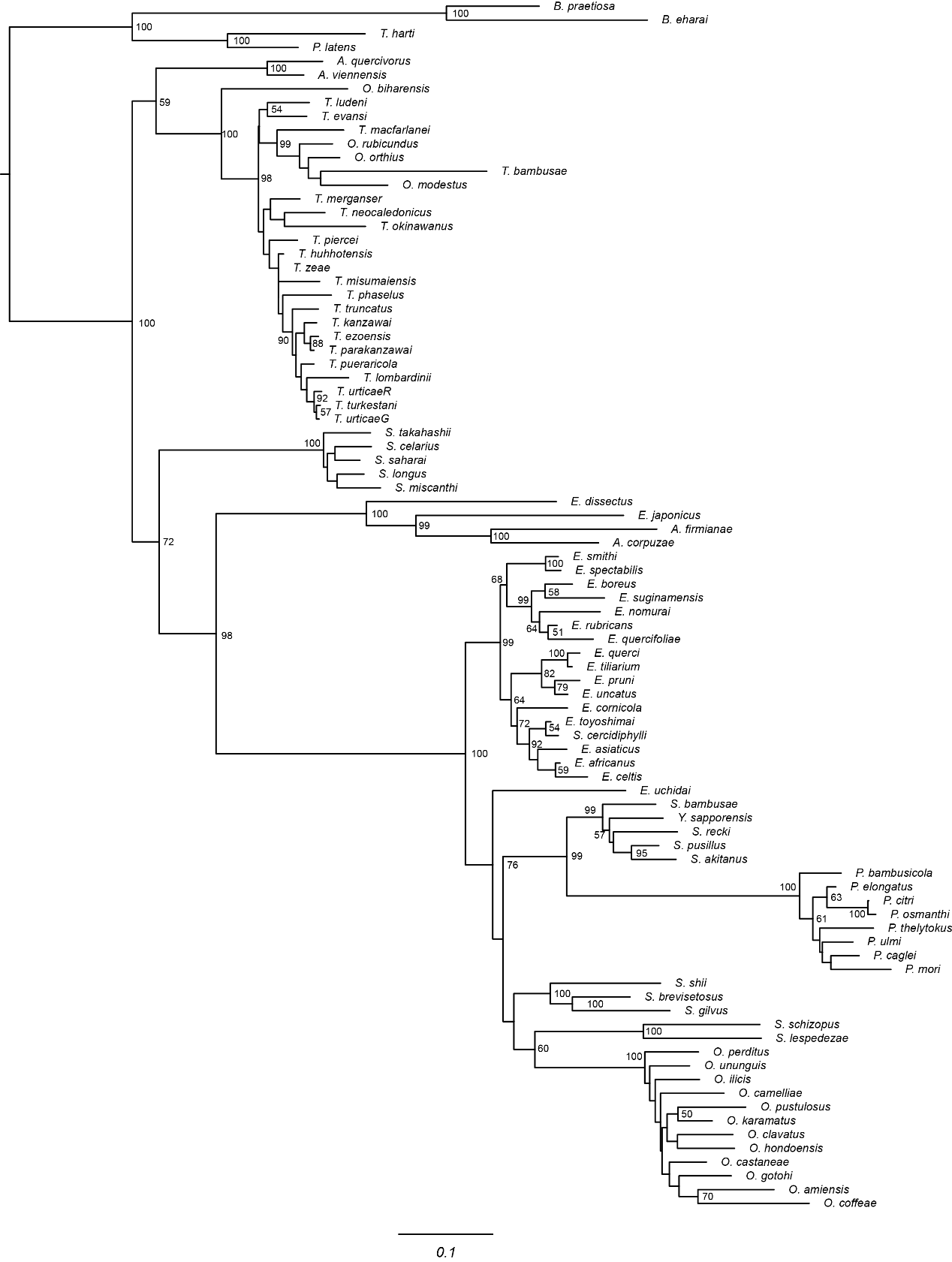


**Fig. S4** Correlations between host specialization and local occurrence in the China dataset (a) and the global dataset (b). p*_ij_* represents the proportion of records of the *j*th mite species found on the *i*th host plant relative to all record numbers, and q*_ij_* represents the proportion of records found for the *j*th mite species on the *i*th host plant relative to all record numbers for the *i*th host. Spearman’s correlations were computed to test for associations. p-values are provided for testing whether correlations are indistinguishable from zero


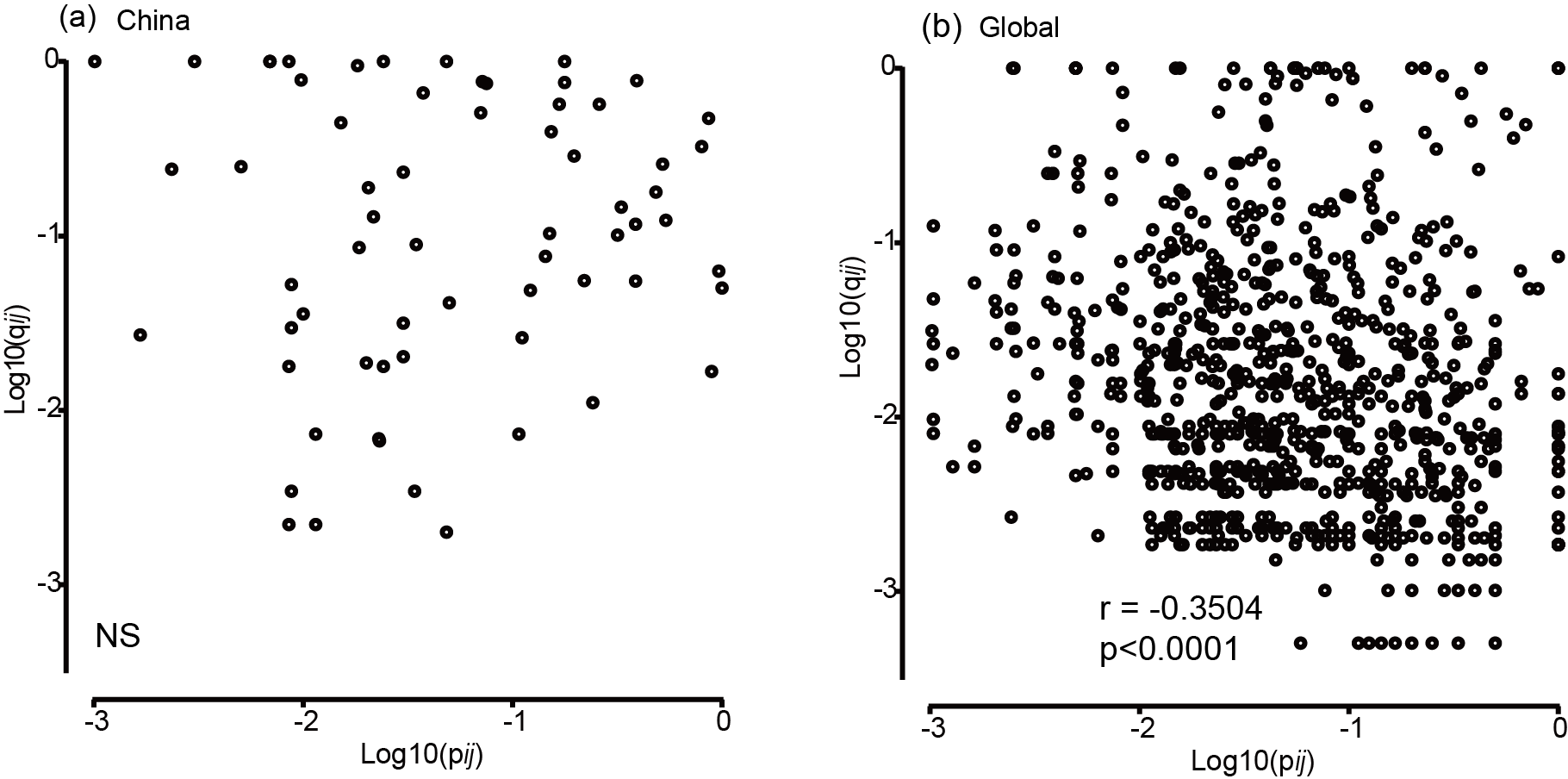

Supplement: Supplementary file 1 — Additional file 1: Table S1. Sample information for samples from China used in this study. Table S2. GenBank accession of sequences used in phylogenetic reconstruction at global scale. Table S3. Relative abundance, host range and distribution of each spider mite species in China. Table S4. Mantel tests of association between genetic distance and species abundance, distribution range and host range. Table S5. Pearson correlations between species occurrence and genetic distance to the focal species of different genera. Figure S1. Bayesian tree with posterior probabilities (a) and RAxML ML tree with bootstrap proportions from 1000 rapid bootstrap replicates (b) for Tetranychus species in China. Figure S2. Bayesian tree with posterior probabilities of 88 species. Figure S3. ML trees based on 1000 rapid bootstrap replicates of 88 species. Figure S4. Correlations between host specialization and local occurrence in the China dataset (a) and the global dataset (b). [file 12862_2019_1548_MOESM1_ESM.docx]
